# Organophosphorus Azoles Incorporating a Tetra-, Penta-, and Hexacoordinated Phosphorus Atom: NMR Spectroscopy and Quantum Chemistry

**DOI:** 10.3390/molecules28020669

**Published:** 2023-01-09

**Authors:** Lyudmila Larina

**Affiliations:** A. E. Favorsky Irkutsk Institute of Chemistry, Siberian Branch of the Russian Academy of Sciences, 1 Favorsky St., 664033 Irkutsk, Russia; larina@irioch.irk.ru

**Keywords:** phosphorylated *N*-vinylazoles, stereochemistry, tetra-, penta-, and hexacoordinated phosphorus atom, phosphorus pentachloride, *E*- and *Z*-isomerism, multinuclear ^1^H, ^13^C, ^31^P NMR spectroscopy, quantum chemistry

## Abstract

The review presents extensive data (from the author’s work and the literature) on the stereochemical structure of functionalized organophosphorus azoles (pyrroles, pyrazoles, imidazoles and benzazoles) and related compounds, using multinuclear ^1^H, ^13^C, ^31^P NMR spectroscopy and quantum chemistry. ^31^P NMR spectroscopy, combined with high-level quantum-chemical calculations, is the most convenient and reliable approach to studying tetra-, penta-, and hexacoordinated phosphorus atoms of phosphorylated *N*-vinylazoles and evaluating their Z/E isomerization.

## 1. Introduction

Phosphorus compounds are attracting considerable research attention due to their wide application in life sciences, agrochemistry and materials science. Over the last decades, the chemistry of organophosphorus compounds is progressing rapidly, owing to their broad utility in the national economy. Diverse biological activity and easy degradation on the simplest non-toxic products allows the organophosphorus compounds (OPC) to be ranked among the top products for chemical plant protection. Phosphorus-containing compounds are reported to be in drugs, insecticides, fungicides, polymer plasticizers and stabilizers, catalysts and components for improvement of lubricating oils’ quality; however, there is more [1,2,3]. Organophosphorus complexes have gained widespread acceptance in science and engineering [4,5,6,7]. However, so far, only a small amount (less than 10%) of the produced phosphorus is spent on the synthesis of organic derivatives. Moreover, organophosphorus compounds are mainly phosphoric acid esters, and only some of them contain one or more P-C bonds. -depth insight into the chemical behavior and biological activity of these systems is impossible without elucidation of structural peculiarities, spectral properties and tautomeric rearrangements. 

A well-known method for the preparation of compounds with a P–C bond is the interaction of phosphorus pentachloride with various nucleophiles (alkenes, alkadienes, alkynes, ethers and esters and tertiary amines including functionalized azoles, etc.). The availability of initial reagents, mild reaction conditions and the ability to vary the structure of the final OPC are advantages of this method. This reaction has not exhausted its synthetic capabilities in terms of the covering of new nucleophiles. Therefore, research in this area still remains a challenge.

The search for new low-waste ways to obtain the phosphorus-containing derivatives of heterocycles, including azoles, through the direct phosphorylation of C–H bonds is a relevant topic. In recent years, phosphorylated azoles have attracted much attention because of the presence of their structural motif in many bioactive naturally occurring and synthetic molecules [8,9,10,11]. Organophosphorus azoles exhibit antiblastic, insecticidal, antihypertensive, hypoglycemic, neurodegenerative and antiexudative activities, among others [10,11,12]. 

Our ongoing research on functionalized azoles and related compounds has been presented in a monograph on nitroazoles [13] and several reviews devoted to the chemistry of five-membered azoles and their benzannelated analogs [14,15,16,17], organosilicon azoles [18], NMR spectroscopy and mass-spectrometry of nitroazoles [19,20], and structure and electronic effects of five-membered nitrogenated heterocycles [21]. Furthermore, tautomerism and the stereochemical structure of functional azoles have been comprehensively researched [22,23,24,25,26]. The problems of prototropy of *NH*-unsubstituted azoles [23] and silylotropy of *N*-silylated analogs [25,26] have been discussed and analyzed in detail using the methods of multinuclear and dynamic NMR spectroscopy and quantum chemistry. The results of the study of the structural peculiarities and tautomerism of functionalized azoles in the solid state using the nuclear quadrupole resonance method [24] summarized.

Azole derivatives are a structural core of numerous biologically important compounds, such as hemoglobin, chlorophyll, antibiotics and vitamin B12. Additionally, they are involved in solar energy fixation, oxygen transfers in natural conditions (in vivo) and other life-sustaining processes. Many medicinal products, plant growth regulators and organic synthesis intermediates are obtained from azoles [13,27].

The stereochemistry of functionalized azoles incorporating phosphorus atoms is a challenging topic in heterocyclic chemistry because the correct interpretation of their chemical behavior and biological activity depends on understanding the factors that determine the stereochemical features and relative stability of their isomers. The study of the structure of phosphorylated *N*-vinilazoles is very important for understanding the reactivity and mechanism of their bioactivity. One of the possible ways of assembling the biologically active organophosphorus and azole moieties in one molecule is the phosphorylation of 1-vinylazoles with phosphorus pentachloride. *N*-vinyl-substituted azoles are usually phosphorylated on a double bond. The lone electron pair (LEP) of the nitrogen atom in 1-vinilazoles, as in vinyl pyrroles, is included in the π-system of the heterocycle. An increase in number of nitrogen atoms in the azole ring, in contrast to pyrroles, enhances the acceptor properties of the heterocycle with respect to the *N*-vinyl moiety. 

The decrease of nucleophilicity of the *N*-vinyl fragment of 1-vinilazoles compared with *N*-vinylpyrroles should reduce the side reactions of chlorination and resinification upon phosphorylation with phosphorus pentachloride. Meanwhile, the presence of pyridine nitrogen atoms in the azole cycles with enhanced nucleophilicity can direct the attack of phosphorus pentachloride in the ring to furnish donor-acceptor complexes containing a hexacoordinated phosphorus atom. Thus, vinyl derivatives of azoles can be the objects of both the donor–acceptor interaction of the azole nucleus with electron-acceptors, and of electrophilic reactions through the double bond of 1-vinylazoles. The reaction course depends on such parameters as the basicity of different sites, the π–electron charge value of pyridine nitrogen, the final charge of pyrrole nitrogen and the polarization of a double bond.

An evaluation of the basicity of 1-vinylazole derivatives shows that imidazole heterocycles have the highest basicity, while that of pyrazoles and 1,2,3-triazoles is reduced, owing to the mutual inductive influence of heteroatoms in the cycle [28,29]. In addition, the basicity of azoles is usually lowered by benzoannelation. The basicity of pyrazoles is much lower than that of imidazoles, and this is a common property of heterocycles with two adjacent nitrogen atoms in the ring (Table 1). 

The phosphorylation reaction involving the vinyl group plays the main role in the interaction of *N*-vinylpyrazoles with phosphorus pentachloride by reducing the basicity of the heterocycle.

The stereochemical structure of phosphorus-containing azoles obtained by the action of phosphorus pentachloride on vinylazoles and related compounds was studied by multinuclear ^1^H, ^13^C, ^15^N, ^31^P, and two-dimensional (2D) NMR spectroscopy [22,30,31,32,33,34].

## 2. The Stereochemical Structure of Phosphorylated *N*-Vinylpyrazoles, *N*-Vinylindazoles and *N*-Vinylbenzotriazoles

**Scheme 1:**
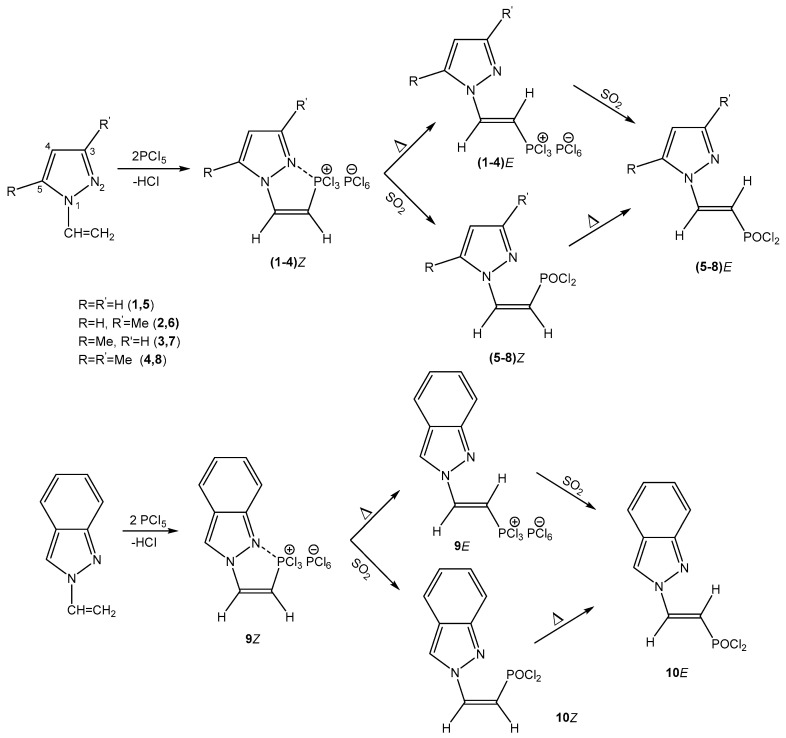
The phosphorylation of 1-vinylpyrazoles and 2-vinylindazole.

1-vinylpyrazoles, 1- and 2-vinylindazoles, and 1-vinylbenzotriazole, having a reduced basicity of the azole moiety, are phosphorylated by phosphorus pentachloride exclusively at the *N*-vinyl group. The phosphorylation of 1-vinylpyrazole, 1-vinyl-substituted 3- or 5-methylpyrazoles, 1-vinyl-3,5-dimethylpyrazole, and 2-vinylindazole creates geometrically favorable conditions (due to the neighboring pyridine nitrogen atom and the *N*-vinyl moiety) for the construction of a donor–acceptor bond between the trichlorophosphonic fragment and the pyridine nitrogen atom of the heterocycle to form *Z*-isomers (**1**–**4**) (Scheme 1) [31,33]. 

The *Z-*configuration of the compounds **1**–**4** is stable at room temperature, while upon heating, a partial *Z-E*-isomerization occurs. The isomerization proceeds much more easily in passing from enaminotrichlorophosphonium hexachlorophosphates (**1**–**4**) to 2-(*N*-pyrazolyl)ethenylphosphonic acid chloroanhydrides (**5**–**8**), and occurs during storage of phosphonic acid chloroanhydrides. This process is facilitated by a decrease in the electron-withdrawing properties of the dichlorophosphoryl moiety compared with the trichlorophosphonic group. Apparently, the presence of hydrogen chloride in the reaction medium promotes *Z*-*E*-isomerization due to the possible reversible hydrochlorination of the double bond of the compounds **5**–**8**. The analogous conversions are observed in the phosphorylation of 2-vinylindazole (**9**, **10**) (Scheme 1). In this case, the pyridine nitrogen atom has a higher basicity than that of 1-vinylindazole, as in the methylated analogs [28] (Table 1).

**Scheme 2:**
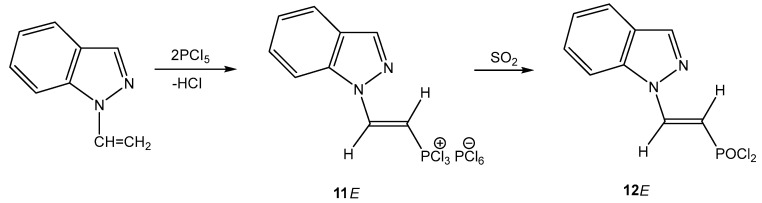
The phosphorylation of 1-vinylindazole.

The reaction of phosphorus pentachloride with low basicity 1-vinylindazole produces exclusively *E*-isomers (**11**, **12**) (Scheme 2).

**Scheme 3:**
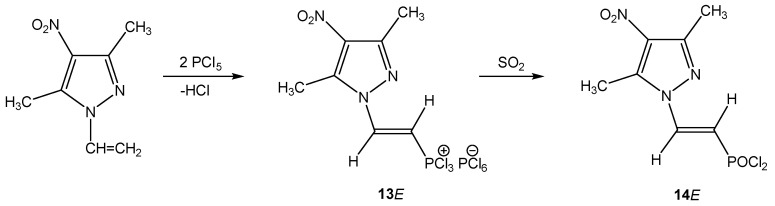
The phosphorylation of 1-vinyl-3,5-dimethyl-4-nitropyrazole.

Meanwhile, the electron-withdrawing action of the nitro group reduces the basicity of the heterocycle; therefore, phosphorylation of 1-vinyl-3,5-dimethyl-4-nitropyrazole (**13, 14**) produces *E*-isomers [22,28,31,33] (Scheme 3).

Apparently, the low basicity of the pyridine nitrogen atom of compounds (**11**, **13**) does not allow the formation of a stable donor–acceptor bond under experimental conditions; therefore, it is not possible to detect the *Z*-isomers of these azoles. 

The donor–acceptor interaction in the *Z*-isomers of the compounds (**1**–**4, 9**) manifests itself so strongly that positive charge is delocalized with the participation of the ring, and the phosphorus atom in the trichlorophosphonic group turns out to be pentacoordinated. In the ^31^P NMR spectra, this leads to the appearance of a signal in the range of −(40–70) ppm that is indicative of significant shielding of the phosphorus nucleus; consequently, there is a shift of positive charge towards the heterocyclic nitrogen atom (Table 2).

**Scheme 4:**
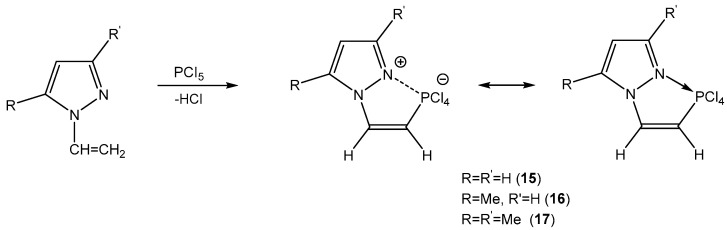
The formation of hexacoordinated *N*-vinylpyrazoles.

It should be noted that the reaction of 1-vinylpyrazoles with phosphorus pentachloride (at a ratio of 1:1) leads to compounds containing a hexacoordinated phosphorus atom (**15**–**17**) (Scheme 4). 

The signals observed in the ^31^P NMR spectra at −(160–200) ppm are assigned to the phosphorus nucleus of the bipolar ions **15**–**17**. The values of coupling constants ^3^*J*_PH_ of approximately 68 Hz and ^3^*J*_HH_ 6 Hz indicate that the *Z*-configuration of the *N*-ethenyl fragment is retained in compounds with a hexacoordinated phosphorus atom (Table 3).

The NMR spectral data of *E*- and *Z*-isomers of 2-(*N*-azolyl)-ethenylphosphonic acid dichloroanhydrides are presented in Table 4 [30,31,33]. *Z*-isomers of alkenylphosphonates show an insignificant high-field shift of the resonance signals of the phosphorus nucleus. The low value of this shift points to the absence of prominent donor–acceptor interactions between the pyridine nitrogen atom and dichlorophosphoryl group of the **5***Z*–**8***Z*, **10***Z*.

**Scheme 5:**
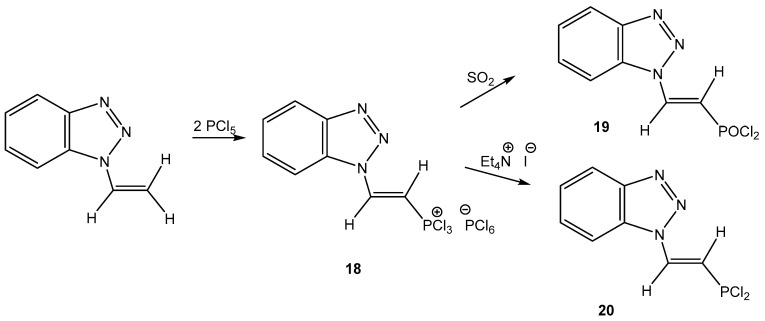
The phosphorylation of 1-vinylbenzotriazole.

The decrease in the nucleophilicity of the adjacent pyridine nitrogen atom in 1-vinylbenzotriazole also increases the competitiveness of the *N*-vinyl group in the reaction with phosphorus pentachloride. 1-vinylbenzotriazole is phosphorylated by phosphorus pentachloride to form benzotriazolyl-*N*-ethenyltrichlorophosphonium hexachlorophosphorate (**18**), which is easily transformed into enaminophosphonic (**19**) and enaminophosphinic (**20**) derivatives [22,30,33] (Scheme 5).

The ^1^H, ^13^C, and ^31^P NMR spectra of the phosphorylation products of 1-vinylbenzotriazole indicate the formation of exclusively *E*-isomers of the phosphorylated **18**–**20** (Table 2 and Table 4). Apparently, this fact is explained by the reduced basicity of the pyridine nitrogen atom (N-2) in 1-vinylbenzotriazole that is typical of 1-alkylsubstitutes (Table 1).

The data of ^31^P NMR spectra show that the reaction mixture after the action of SO_2_ on hexachlorophosphorates **1**–**4**, **9** contains, along with the major compounds **5**–**8**, **10**, hydrochlorination products **21**–**25** in minor amounts (Table 5) [33].

The multiple signals of the phosphorus atom of compounds **21**–**25** appear in a lower field than the signals of the compounds **5**–**8**, **10**. In this case, small variations in **21**–**25** insignificantly change the chemical shifts (33–35 ppm) and coupling constants (Table 5). 

**Scheme 6:**
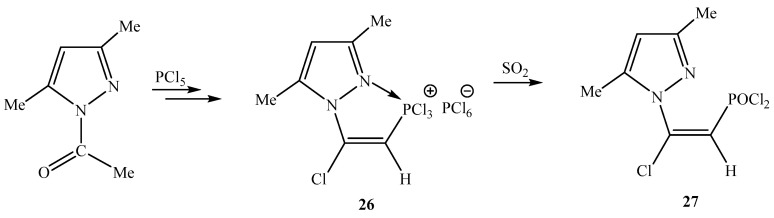
The phosphorylation of 1-acetyl-3,5-dimethylpyrazole.

The phosphorylation of 1-acetyl-3,5-dimethylpyrazole furnishes pyrazolium hexachlorophosphorate **26**—2-chloro-2-(3,5-dimethylpyrazolyl)ethenyltrichlorophosphonium—in the form of the *E*-isomer (Scheme 6) [31].

In the ^31^P NMR spectrum of **26**, a doublet at –52.7 ppm (^2^*J*_PH_=47 Hz) corresponds to the PCl_3_^+^ fragment. Complex salt **26** is easily converted into 2-chloro-2-(3,5-dimethylpyrazolyl)ethenylphosphonic acid dichloride (**27**). In the ^31^P NMR spectrum of **27**, δ 19.9 ppm with ^2^*J*_PH_ = 21 Hz.

## 3. Computation of ^31^P NMR Chemical Shifts of Tetra-, Penta- and Hexacoordinated Phosphorus Atom in Phosphorylated *N*-Vinylpyrazoles

Advances in the theoretical methods used to calculate NMR parameters have led to a major breakthrough in NMR computational applications for structural studies of organic molecules including organophosphates. This has stimulated top-level investigations of conformational and configurational manifestations (tasks) in molecular, intramolecular and intermolecular interactions, along with spectral assignment and structural explanation. Key attention is paid to the study of ^31^P NMR chemical shifts that allow us to shed a light on various structural aspects of organophosphorus compounds. The range of phosphorus chemical shifts is known to exceed 500 ppm, which evidences the importance of ^31^P NMR spectroscopy for structural studies of organophosphorus compounds [35,36,37,38]. 

One of the most intriguing features of ^31^P NMR spectroscopy relates to the effects originating from the intra- and intermolecular coordination of phosphorus with heteroatoms containing a lone electron pair. In this case, the coordination effect can reach significant values comparable to the full range of changes in the chemical shifts of phosphorus. So, for example, phosphorus pentachloride, a well-known electrophilic phosphorylating agent, both in the crystalline state and in polar solvents dissociates into ions, and in non-polar solvents exists in a pentacoordinated state; meanwhile, the chemical shift of phosphorus varies from –300 to +80 depending on the degree of coordination (Equation (1)) [33] (Table 6).

(1) $${2\mathrm{PCl}}_{5}\;⇌{\;\mathrm{PCl}}_{4}^{+}{\;+\;\mathrm{PCl}}_{6}^{\;-}$$

Indeed, an increase in the coordination number of phosphorus from 3 to 6 leads to a dramatic shielding of the ^31^P nucleus from approximately +200 to −300 ppm [39]. The theoretical study of this interesting effect seems to be a challenging task of great practical importance. In addition to the available experimental data, modern quantum chemical calculations allow a deeper understanding of the nature of the relationship between the ^31^P NMR chemical shifts and the structure of phosphoroorganic compounds with different coordination numbers. 

**Scheme 7:**
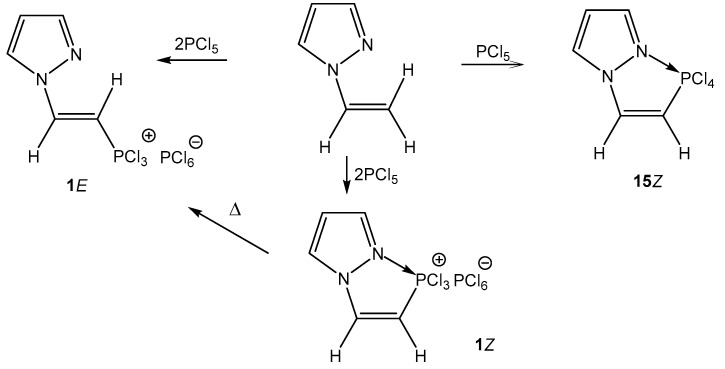
Tetra-, penta- and hexacoordinated phosphorus atoms in *N*-vinylpyrazoles.

The effect of intramolecular coordination on ^31^P nuclear shielding in the series of tetracoordinated, pentacoordinated and hexacoordinated *N*-vinylpyrazoles represented by 2-(1-pyrazolyl)ethenyltrichlorophosphonium hexachlorophosphorate (**1**) and 2-(1-pyrazolyl)ethenyltetrachlorophosphonium (**15**) has been examined [33,37,40,41]. Phosphorylated pyrazole **1** has two configurational isomers, **1***E* and **1***Z*, containing tetracoordinate and pentacoordinate phosphorus atoms, respectively; meanwhile, compound **15** is isolated only as the *Z*-isomer, **15***Z*, the latter having a hexacoordinate phosphorus atom (Scheme 7).

The *Z* configuration of isomer **1** is stable at room temperature, and the *Z*-*E*-isomerization occurs only upon heating. The presence of hydrogen chloride in the reaction medium facilitates *Z*-*E*-isomerization due to the possibility of reversible hydrochlorination of the double bond of this compound. 

The obtained experimental data show that in the ^31^P NMR spectrum of compound **1***Z*, a signal is observed at −55.2 ppm, which, in comparison with the chemical shift of its configurational isomer **1***E* (+94.4 ppm), indicates a significant shielding of the phosphorus nucleus due to the possible appearance of a donor–acceptor interaction between the phosphorus atom of the chlorophosphonium group and the pyridine nitrogen atom, that is, a structure with a pentacoordinated phosphorus atom. Such a donor–acceptor interaction in **15***Z* contributes to the formation of a structure with a hexacoordinated phosphorus atom (δ^31^P = −216.2 ppm). To confirm the conclusions about intramolecular coordination obtained from the analysis of experimental data, ab initio calculations of the shielding constant of the phosphorus nucleus in these compounds were carried out. 

**Figure 1:**
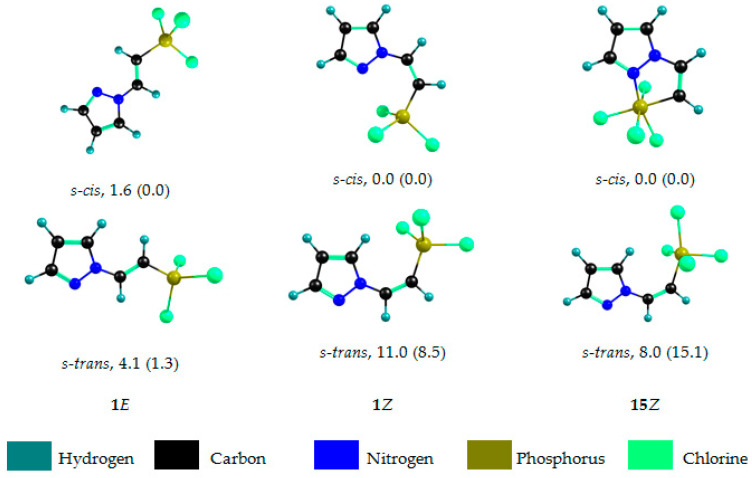
Predominant conformations and relative total energies (kcal/mol) of the *s*-*cis* and *s*-*trans* conformers of compounds **1** and **15** calculated by the B3LYP/6-311G(d,p) method for the gas phase and with taking into account the solvation effects within the polarizable continuum model (in parentheses).

The shielding constants were calculated for the preferred conformations of azoles **1** and **15** localized at the B3LYP/6-311G(d,p) level for the gas phase, alongside using the polarizable continuum model (PCM) to take into account effect of the medium, in this case nitromethane. The structures of the most stable conformers and relative energies of compounds **1** and **15** are shown in Figure 1 [40,41].

According to the results of the theoretical conformational analysis at the B3LYP/6-311G(d,p) level, internal rotation around the N-C bond in **1***E*, **1***Z* and **15***Z* leads to the existence of two stable rotational conformations, *s-cis* and *s-trans*, with dihedral angle values NNCC = 0° and 180°, respectively. The only exception is the *s-trans* conformer of **15***Z*, for which this angle is 170°. This is explained by steric interactions between the bulky group of PCl_4_ and the pyrazole ring.

Characteristically, for compounds **1***Z* and **15***Z*, both in the gas phase and taking into account the solvent field, the *s-cis* conformation is the most favorable, the energy of which is much lower than the corresponding values for the *s-trans* conformers, which leads to almost 100% content of the *s-cis*-form. The relative stability of the *E-* and *Z-*isomers of **1** (*s-cis* conformers) depends on the solvent’s nature. In the gas phase, the *Z-*isomer is more stable than the *E-*isomer by 1.6 kcal/mol, whereas calculations that take into account the solvent field gave almost similar energies for both isomers. Thus, the possibility for experimentally observable *Z*–*E-*isomerization of salt **1** at elevated temperature was theoretically substantiated.

Fundamentally important information about the nature of intramolecular coordination in phosphorylated *N*-vinylpyrazoles can be obtained from calculations of the shielding constant of the ^31^P nucleus, which were carried out in terms of the density functional theory (DFT) by the B3LYP method [42,43] within the framework of the gradient-invariant atomic orbital (GIAO) approach [44]. It has been shown earlier [37,45] that relativistic effects are of decisive importance in the calculation of δ^31^P for molecules containing elements of the third or more periods. For example, in the series of phosphines (CH_3_)_3_PX, where X = O, S, and Se for trimethylphosphine sulfide and trimethylphosphine selenide, the value of the spin–orbital contribution reaches 10 and 40 ppm, respectively. On the other hand, for PCl_4_^+^ cation, the spin–orbital interaction also results in a high-field shift of the phosphorus resonance signal by 40–50 ppm. These data indicate that the calculation of the ^31^P NMR chemical shifts in the chlorophosphonium group of compounds **1** and **15** requires allowance for the spin–orbital interaction.

The contribution of spin–orbital interaction is taken into account in terms of the zero-order regular approximation (ZORA) for the relativistic effects [46,47] implemented in the ADF 2009 software package [48]. As basis sets for nonrelativistic calculations, the triple-split Kutzelnigg IGLO-III was used [49], which showed the best results in calculating ^31^P NMR chemical shifts in phosphines and phosphine oxides [50]. For calculations taking into account the spin–orbit interaction, a double-split Slater-type basis set DZP [51,52] was employed. The method for calculating the relativistic values of the ^31^P NMR chemical shifts within the framework of the ZORA approach included the calculation of the nonrelativistic value of the chemical shift δNR and the spin–orbit contribution δSO, the sum of which leads to the relativistic value δR. The medium was taken into account in the framework of the COSMO continuum model [53,54]. Theoretical values of ^31^P NMR chemical shifts δcalc (ppm) are given relative to 75% H_3_PO_4_ aqueous solution, recalculated according to Equation (2) and given in [55]:

(2) $$\delta_{\mathrm{calc}}\;{=\sigma }_{\mathrm{calc}}\left({\mathrm{PH}}_{3}\right)-{\;\sigma }_{\mathrm{calc}}\;-\;266.1$$

Here, σ_calc_ is the absolute shielding constant of the phosphorus nucleus of the studied phosphine, and σ_calc_ (PH_3_) is the absolute shielding constant of the phosphorus nucleus PH_3_, calculated at the same level of theory.

The calculated ^31^P NMR chemical shift values for the *s-cis* and *s-trans* conformations of compounds **1** and **15** are shown in Table 7. The results of the GIAO-B3LYP/DZP calculations are in good agreement with the experimental values for the respective preferred conformations of compounds **1***E* and **15***Z*. The calculation error does not exceed 25 ppm, both in the case of calculation in the gas phase and with allowance for the effects of the medium. However, in the case of the *s-cis* conformer **1***Z*, the calculated value of the ^31^P NMR chemical shift by 160 ppm exceeds the experimental value, which indicates that in the gas phase and under conditions of nonspecific solvation, the formation of the intramolecular N→P coordination bond does not occur. Indeed, geometry optimization of the *s-cis*-conformer does not lead to a change in the distance between the phosphorus and nitrogen atoms.

**Figure 2:**
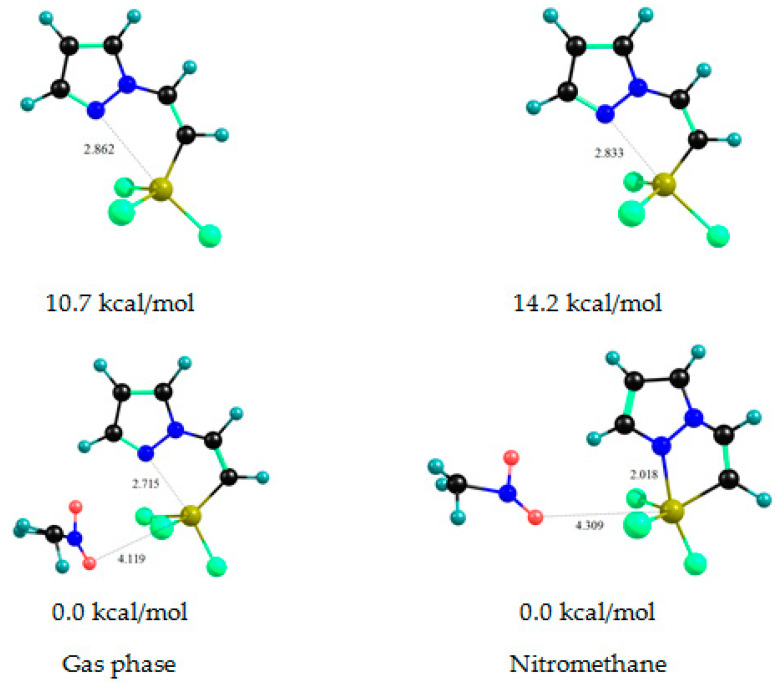
Preferred conformations and relative total energies (kcal/mol) of compound **1***Z* and its complex with nitromethane molecule, calculated by the B3LYP/6-311G(d,p) method and taking into account the solvation effects within the polarizable continuum model. Bond lengths are given in Å.

The fundamental moment in the formation of an intramolecular coordination bond in compound **1***Z* is the influence of the solvent, in this case nitromethane. In this regard, an additional theoretical study of the structure of the complex of compound **1***Z* with one nitromethane molecule was carried out, which included optimization of the geometry in the gas phase and in the solvent medium (Figure 2). 

The formation of the **1***Z* complex with a nitromethane molecule reduces the energy of the system, while in the gas phase, an intramolecular dative bond between the phosphorus atom and the pyridine-type nitrogen atom is not formed. The P–N interatomic distance shortens to 2.018 Å upon passing from the gas phase to the solution, which indicates the presence of a donor–acceptor interaction between the phosphorus and nitrogen atoms, and the stabilization of this structure by the solvent. Calculations of the ^31^P NMR chemical shift for this complex indeed indicate a significant low-frequency shift of the phosphorus resonance signal due to the formation of an intramolecular dative bond. Exceeding the calculated value of the ^31^P NMR chemical shift by 45 ppm may be due to the insufficient theoretical level of the used solvation model. Perhaps, for a correct description of intramolecular coordination bonding, it is necessary to use a larger number of solvent molecules to model the solvate shell [40,41]. 

The change in the phosphorus signal can be assessed by comparing the δP values for the *s*-*cis* and *s*-*trans* conformers of the respective isomers. Both **1***Z* conformers are characterized by almost the same δP values, which may be due to the lack of coordination. Upon transition to the **1***Z* complex with nitromethane, the formation of an intramolecular dative N→P bond leads to a low-frequency shift of the phosphorus signal by 120 ppm. The coordination effect for compound **15***Z* is estimated at 155 ppm for the gas phase and 170 ppm for solution. The spin–orbit interaction makes a significant contribution to the overall coordination shift of the phosphorus signal. The formation of a dative bond in **1***Z* and **15***Z* is accompanied by an increase in the spin–orbit contribution by 9.5 and 25 ppm, respectively; these values are 8 and 15% of the coordination induced shift.

Thus, the results of this study indicate the need to take into account the relativistic corrections and medium effects when calculating the shielding constants for phosphorus nuclei. 

## 4. The Structural Features of Molecular Complexes of Vinylazoles with Phosphorus Pentachloride 

*N*-vinylpyrazoles, as well as the corresponding indazoles and benzotriazoles, are phosphorylated at the double bond by phosphorus pentachloride to provide azoles containing the tetra-, penta-, or hexacoordinated phosphorus atom. *N*-vinylimidazoles or *N*-vinylbenzimidazoles, having highly basic isolated (separated) pyridine nitrogen atoms, interact in the cycle with phosphorus pentachloride in a different way. Donor–acceptor complexes in these reactions are initially generated. 

**Scheme 8:**
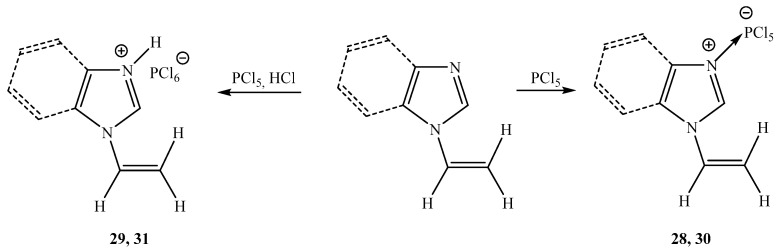
The phosphorylation of *N*-vinylimidazole and *N*-vinylbenzimidazole.

The reaction of *N*-vinylimidazole with phosphorus pentachloride, even with its excess, produces the donor–acceptor complex **28** with the involvement (participation) of the pyridine nitrogen atom [32]. Consequently, the electron-withdrawing properties of the ring are enhanced and the nucleophilicity of the *N*-vinyl group decreases, which hinders its phosphorylation. The interaction of the vinylimidazole with the phosphorus pentachloride in benzene solution saturated by hydrogen chloride gives the complex **29,** containing the protonated nitrogen atom in the position 3 (Scheme 8). 

Analogously, the action of PCl_5_ on *N*-vinylbenzimidazole provides a donor–acceptor complex **30**, whereas 3-*H*-1-vinylbenzimidazolinium hexachlorophosphate **31** is formed in the presence of hydrogen chloride. ^1^H, ^13^C, and ^31^P NMR data of complexes **28**–**31** are shown in Table 8.

The presence of three proton signals of *N*-vinyl groups in the ^1^H NMR spectra of compounds (**28**–**31**) in nitromethane indicates that the vinyl group does not participate in the phosphorylation reaction. The signals recorded in the region −(258–296) ppm in the ^31^P NMR spectra are typical for the hexacoordinated phosphorus atom.

**Scheme 9:**
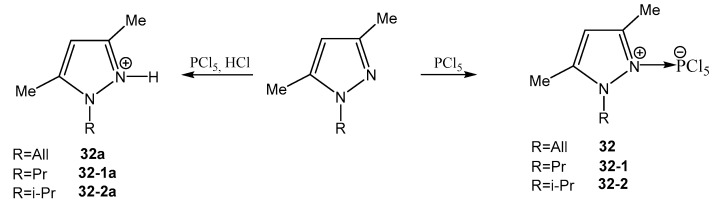
The phosphorylation of 1-allyl-, 1-propyl- and 1-isopropyl-3,5-dimethylpyrazole.

1-allyl-3,5-dimethylpyrazole [32] and the corresponding 1-propyl and 1-isopropyl derivatives interact with phosphorus pentachloride exclusively with the participation of the pyridine nitrogen atom of heterocycle. The presence of a pyridine nitrogen atom with increased nucleophilicity in the azole ring directs the attack of phosphorus pentachloride into the heterocycle to form the donor–acceptor complexes **32, 32**–**1** and **32**–**2** (Scheme 9). 

The phosphorus singlet signals in the ^31^P NMR spectra of compounds **32**, **32**–**1** and **32**–**2** appear in the −(260–264) ppm region and characterize the hexacoordinated phosphorus atom (Table 8).

**Scheme 10:**
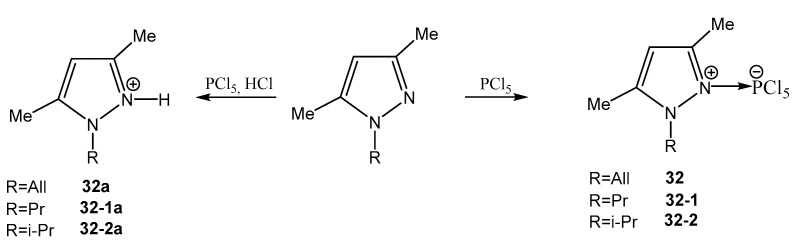
The phosphorylation of 1-benzyl-3-vinyl-5-chloropyrazole and 1-benzyl-3-isopropenyl-5-chloropyrazole.

C-alkenylazoles, like *N*-vinilazoles, are phosphorylated at the double bond. It has been established that 1-benzyl-3-vinyl-5-chloropyrazole and 1-benzyl-3-isopropenyl-5-chloropyrazole are phosphorylated by phosphorus pentachloride at the vinyl and isopropenyl groups to produce organyl trichlorophosphonium hexachlorophosphonium **33** and **34** (Scheme 10) [32].

Hexachlorophosphorates **33**, **34,** under the action of acetone in diethyl ether, are easily converted into phosphonic acid dichlorides **35**, **36** (Table 8).

## 5. Quantum-Chemical Calculations of ^31^P NMR Chemical Shifts of Molecular Complexes of Azoles

Theoretical examination of molecular complexes of phosphorus pentachloride with 1-vinylimidazole (**28**) and 1-allyl-3,5-dimethylpyrazole (**32**) have been carried out [41,56]. The phosphorus atom of the chlorophosphonium group can form a donor–acceptor bond with a pyridine nitrogen atom containing the lone electron pair (i.e., an sp_2_-hybridized nitrogen atom). As a result, the coordination number of phosphorus atom increases to 5 or 6, and its resonant signal in the ^31^P NMR spectrum is shifted up-field. In addition, the presence in the heterocycle of nitrogen atoms of the pyridine type having increased nucleophilicity makes it possible to direct the attack of phosphorus pentachloride not to the double bond of the vinyl group, but to the heterocycle, with the formation of the corresponding intermolecular donor–acceptor complexes **28** and **32** [32,34,56] (Scheme 8 and Scheme 9). The ^31^P NMR spectra of **28** and **32** contain a singlet in the region δ^31^P from −258 to −296 ppm, which is typical of six-coordinate phosphorus atoms. Quantum chemical calculations of ^31^P NMR chemical shifts in **28** and **32** were carried out for the gas phase and taking into account the effect of the solvent in order to evaluate the effect of intermolecular coordination on the ^31^P shielding constants (chemical shifts) (Table 9).

**Figure 3:**
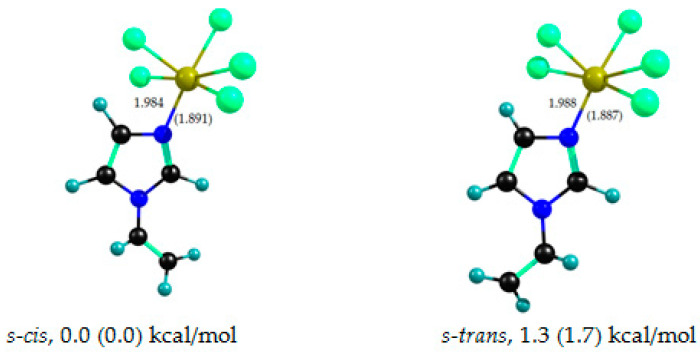
Predominant conformations of the complex of *N*-vinylimidazole with PCl_5_ optimized by the B3LYP/6-311G** method, and their relative energies. Bond lengths are given in Å. The relative energies and bond lengths, calculated taking into account the solvent effect (nitromethane) within the PCM, are given in parentheses.

**Figure 4:**
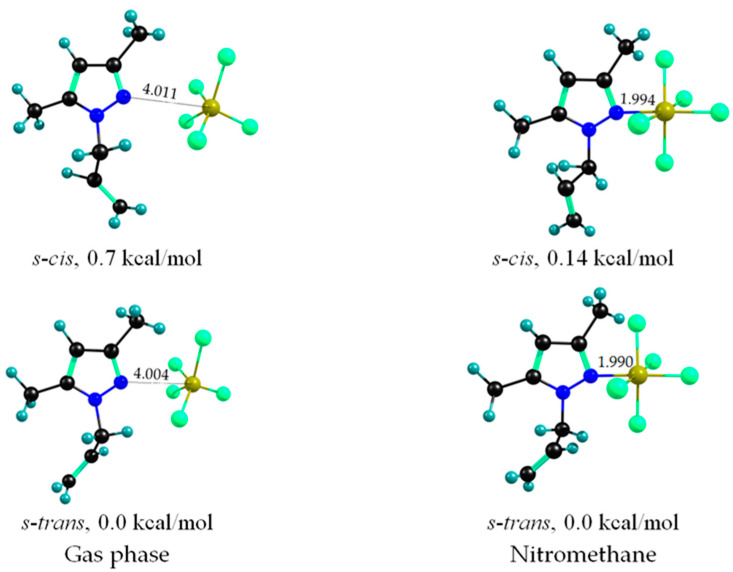
Predominant conformations of the complex of 1-allyl-3,5-dimethylpyrazole with PCl_5_ **32** calculated by the B3LYP/6-311G(d,p) method, and their relative energies. Solvent (nitromethane) effect was taken into account within the PCM. Interatomic distances are given in Å.

The phosphorus shielding constants were calculated at the GIAO-B3LYP/DZP level of theory, taking into account the spin–orbital relativistic interaction in terms of the ZORA [46,47]. The theoretical ^31^P chemical shifts δ_calc_ (ppm) were recalculated according to Equation (1) and are given relative to 85% aqueous H_3_PO_4_ [55]. All calculations were performed for molecular complexes, with geometric parameters optimized in terms of the density functional theory using B3LYP hybrid functional and a 6-311G(d,p) basis set for the gas phase, and with account taken of the solvent field (nitromethane). The solvent effect was simulated according to the polarizable continuum model (PCM); nitromethane was selected as a solvent, and the experimental ^31^P NMR spectra were recorded in this solvent. The most favorable conformations of complexes **28** and **32** and their relative energies are shown in Figure 3 and Figure 4. 

The calculated ^31^P NMR chemical shifts of complex **28** both in the gas phase and in solution are in good agreement with the experimental values (see Table 10). Deviations do not exceed 12 ppm, which may indicate the complexation of vinylimidazole with phosphorus pentachloride. The theoretical conformational analysis data also evidence the generation of an N→P bond, because the distance between the pyridine nitrogen atom and the phosphorus atom is not higher than 2 Å, and the configuration of the phosphorus atom is modified to be octahedral, which is typical for *sp*^3^*d*^2^ hybridization. It should also be noted that consideration of the solvent effect in the geometry optimization leads to a shortening of the P–N distance in both conformers **28** by about 0.1 Å, which eventually favors more appropriate calculation of the ^31^P chemical shift. 

The calculated ^31^P chemical shift of complex **32** in the gas phase (−63 ppm) differs significantly from the experimental value (−264.3 ppm) and corresponds to the range typical of five-coordinate phosphorus atoms, about −80 ppm (Table 9). Based on calculations for the gas phase by means of B3LYP/6-311G(d,p), the distance between pyridine nitrogen atoms and phosphorus atoms exceeds 4 Å, while the PCl_5_ molecule retains the bipyramidal configuration. Thus, the formation of a molecular complex between 1-allyl-3,5-dimethylpyrazole and phosphorus pentachloride does not occur in the gas phase. Taking into account the influence of the solvent (nitromethane), the P–N distance shortens to 2 Å, and (as in complex **28**) the steric configuration of the PCl_5_ molecule transforms from trigonal bipyramidal to octahedral. This is accompanied by an increase in the calculated ^31^P NMR chemical shift (up to −282.5 ppm) in the energetically most favorable *s-trans* conformer.

Assessment of the ^31^P chemical shifts of the starting phosphorus pentachloride and its molecular complexes with azoles shows that the creation of the N→P dative bond is attended by a low-frequency shift of the phosphorus signal by 220 ppm, of which 60 ppm is due to the contribution of the spin–orbit interaction [56].

The effect of the solvent must be taken into consideration when optimizing the geometric parameters and calculating the ^31^P NMR chemical shifts for intra- and intermolecular complexes formed with the participation of a phosphorus atom and a nitrogen atom in heteroaromatic systems. As in the case of intramolecular coordination in the **15***Z* isomer, allowance for the effect of the solvent is of decisive importance because the solvent is responsible for the stabilization of such complexes. Accounting for relativistic effects is essential for the accuracy of predicting ^31^P chemical shifts. The contribution of the spin–orbit interaction is about 200 ppm, and neglecting this contribution would lead to incorrect predictions.

## 6. Theoretical ^31^P NMR Chemical Shifts of Pyrazolylphosphine and Related Compounds

A similar theoretical approach was applied to the study of ^31^P NMR chemical shifts and the stereochemical behavior of phosphonic acid dichlorides containing the POCl_2_ group at the C=C and C=N double bonds, i.e., 2-(pyrazolyl)ethenylphosphonic acid dichloride (**5**) and the related compounds, *N*-dichlorophosphonylimin obenzoic acid chloride (**37**), *N*′-dichlorophosphoryl-*N*,*N*-dimethylchloroformamidine (**38**). These were prepared by the phosphorylation of *N*,*N*-dimethylurea with phosphorus pentachloride [57] and 1-trichlorophosphazo-1-chloro-2-dichlorophosphoryl-2-azaethene (**39**) obtained by the interaction of urea with phosphorus pentachloride [58]. The ^31^P NMR chemical shifts were also calculated by the GIAO-B3LYP-ZORA/DZP for the predominant conformation of compounds **5, 37**–**39** for the gas phase (Table 10) [59]. The theoretical ^31^P NMR chemical shift values are also recalculated by the Equation (2). 

Analysis of the NMR spectra shows that compound **5** existed in the form of two isomers which were assigned by the measurement of spin–spin coupling constants ^3^*J*_PH_ that sharply differed between the configurational isomers with respect to the double bond [33]. In addition to these data, it was interesting to test the possibility of using ^31^P NMR chemical shifts for the configuration assignment of compounds containing a double bond C=C linked to a phosphorus atom. Therefore, we calculated the ^31^P NMR chemical shifts and performed the theoretical conformational analysis of these compounds in order to explore the influence of configuration and the conformational effects on the ^31^P NMR spectra. The results of these calculations, including the localization of the prevailing conformers, the calculation of the relative energies (*E*_rel_) and ^31^P NMR chemical shifts of compound **5,** are presented in Table 11. 

The internal rotation in both isomers **5** around the P–N bond as well as the rotation of the pyrazole ring around the C–N bond is allowed. The calculations show that four rotational conformers are localized for each isomer (Table 11). Theoretical data reveal that the *E*-isomer **5** is energetically more favorable than the *Z*-isomer in all probable conformations, as evidenced by the experimentally observed isomerization of the **5***Z-*isomer into the *E*-isomer during heating or storage. The most stable conformations of the *E*-isomer are *s-cis-s-cis* and *s-trans-s-cis,* with an energy difference of 1.4 kcal/mol. The energy of the other two conformations, *s-cis-gauche* and *s-trans-gauche*, is much higher and, therefore, they are practically absent in the equilibrium mixture. The equilibrium in the **5***Z*-isomer is almost completely shifted to the *s-cis-gauche* conformation due to steric hindrances preventing the existence of other conformers (Table 11).

The ^31^P chemical shift values in the *E*-isomer **5** change within a fairly narrow range of 29–33 ppm, indicating the absence of any electronic effects at conformational variations. However, the range of ^31^P NMR chemical shift variation in the *Z*-isomer is much larger, at 16–33 ppm. In the energetically more favorable *s-cis-gauche* and *s-cis-s-trans* conformers, a low-frequency shift seems to be due to the influence of the lone electron pair of the pyridine nitrogen atom. Nevertheless, in the *s-trans-s-cis*-conformer, where this interaction is absent, the chemical shift is the same as in the *E*-isomer, indicating an insignificant effect of the configuration change at the double bond on the ^31^P NMR chemical shifts (Table 11) [59].

Compounds **37**–**39** exist in solution as individual isomers, the configuration assignment of which is a rather complicated problem and cannot be directly solved on the basis of NMR spectroscopy data. For this purpose, theoretical conformational analysis and calculation of the ^31^P NMR chemical shifts were performed for each of the conformers of both isomers **37**–**39** containing the C=N–POCl_2_ fragment (Table 12, Table 13 and Table 14, respectively).

The results of conformational analysis indicate that the *Z*-isomers of compounds **37**–**39** (Table 12, Table 13 and Table 14) are more favorable in terms of energy, and their energy is 2–9 kcal/mol lower than that of the *E*-isomers. The internal rotation around the N–P bond in the *Z*-isomers **37**–**39** points to the existence of three stable rotational conformers: *s-cis*, *s-trans*, and *gauche*. The three conformers are close in energy; however, in all cases the *gauche* form predominates, while the relative energy of *s-cis* and *s-trans* conformers is about 0.7 kcal/mol. This evidences almost equal content of these conformers in an equilibrium mixture. In the *E*-isomers of compounds **37**–**39**, strong steric strains between the substituent and the bulky POCl_2_ group were found. The *gauche* conformer is also the most stable in the *E*-isomers. However, in *E*-isomer **37**, all three possible conformers can exist: *s-cis*, *s-trans*, and *gauche* (Table 12), and steric strain in *s-cis* and *s-trans* conformers leads to a significant deviation of the benzene ring from the double bond plane.

Conformational transitions in compound **39** (Table 14) occur due to the rotation of the N=PCl_3_ fragment around C–N bond, which delivers two rotational conformations, *s-cis* and *s-trans*, while the *s-cis* forms in the *Z*-isomer are more favorable than the corresponding *s-trans* conformations by 3–5 kcal/mol. According to calculations in the *E*-isomer, only *s-trans* conformations with *gauche* and *s-trans*-orientations of the P=O bond can exist.

A similar influence on ^31^P NMR chemical shifts is detected in compounds **37**–**39**. Both isomers of compounds **37**–**39** in *s-cis-* and *gauche-*forms have almost identical ^31^P NMR chemical shifts. The only exclusions are *s-cis*-conformer **37***E* and *gauche*-conformer **38***E* which are characterized by a low-frequency shift of the resonance ^31^P signal of about 10–15 ppm; this is perhaps attributable to the changes in the bond angles and bond lengths due to steric interactions compared with energetically favorable conformations. The up-field displacement of the phosphorus chemical shift in *s-trans*-conformations by approximately 15 ppm seems to be due to the formation of the C=N–P=O conjugated system, which may indicate a decrease in the length of the ordinary N–P bond by 0.2 Å compared with the *s-cis-* and *gauche-*forms. An analogous effect was observed in compound **39***Z* during rotation around the C–N bond. At the *s-trans*-location of the P=N and C=N bonds, the position of the ^31^P NMR signal has a low-frequency shift of 30 ppm, which can be explained by the conjugation effect. 

Thus, there is good agreement between the theoretical and experimental ^31^P NMR chemical shift values in the corresponding compounds in their prevailing conformations. In this case, taking into account the presence of the conformational equilibrium, the chemical shifts must be averaged with consideration of the occupancy of the rotational conformations. The theoretical ^31^P chemical shift values averaged over all localized conformations of *E*- and *Z*-isomers of compounds **5, 37**–**39** are comparable with the experimental data (Table 10) [59]. 

## 7. The Stereochemical Structure of Phosphorylated Pyrroles and Their Annulated Analogs

1-vinylpyrroles and their annulated analogs, indoles and carbazoles have also been phosphorylated by the reaction with phosphorus pentachloride to provide various phosphorylation products both at the vinyl group and at the pyrrole cycle [60,61,62,63]. The introduction of a phosphorus-containing fragment into the pyrrole rings is a structural motif of bioactive natural products and pharmaceutical drugs [64,65,66,67]. It allows us to expect the appearance of unusual biological activity in the phosphorylated pyrroles. The natural compound psilocybin, dimethyltryptamine-4-phosphate, is known to have strong hallucinogenic activity [62]. 

**Scheme 11:**
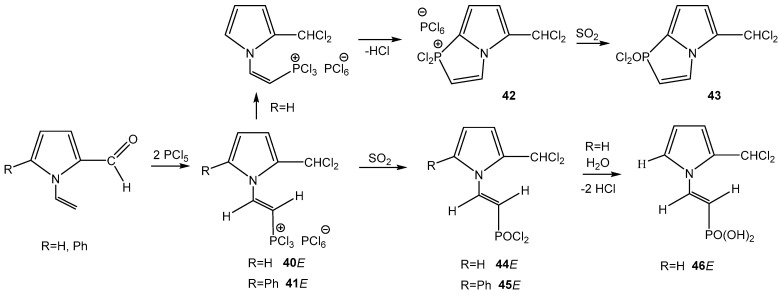
The phosphorylation of 1-vinylpyrrole-2-carbaldehydes.

1-vinylpyrrole-2-carbaldehydes react stereoselectively with an excess of phosphorus pentachloride involving both formyl and vinyl groups to produce *E*-2-(2-dichloromethylpyrrol-1-yl)vinylphosphonium hexachlorophosphates **40** and **41** (Scheme 11, Table 15).

The ^31^P NMR signals of salts **40**, **41** detected at –296 ppm and 89.7–92.1 ppm are assigned to PCl_6_^−^ and PCl_3_^+^, correspondently, wherein the signals of PCl_3_^+^ cations appear as doublets of doublets, ^2^*J*_PH_ = 13 Hz and ^3^*J*_PH_ = 33 Hz. This indicates the *E*-configuration of the vinyl fragment. Besides which, the ^31^P NMR spectrum of salt **40** in MeNO_2_ has multiple signals at 66.6 ppm with ^2^*J*_PH_ = 7.9 Hz and ^3^*J*_PH_ = 25.7 Hz. This can be attributable to 3,3-dichloro-6-(dichloromethyl)-1H-1k5-pyrrolo-[1,2-a]-1,3-azaphospholidinium hexachlorophosphate **42** (approx. 8–10%, ^31^P NMR) [68]. The latter is most likely produced via electrophilic cyclization of the *Z*-isomer **40** (Scheme 11).

The ^31^P NMR signals of *E*-2-(2-dichloromethyl-1-pyrrol-1-yl)vinylphosphonyl dichlorides **44**, **45** are in the region of 31.9–34.4 ppm (dd, *J*_PH_ = 23.1–24.8 Hz) and belong to the POCl_2_ fragment. The ^31^P NMR spectrum of dichloride **44** exhibits a low intensity signal at 20.7 ppm, which evidences the presence of the corresponding cyclic phosphinyl chloride **43** (6%, 31P NMR). In addition, phosphonyl chloride **44** is readily transformed to the corresponding phosphonic acid **46**. It should be noted that the values of the ^1^H and ^13^C NMR chemical shifts of the pyrrole ring atoms in the considered phosphorylated series are weakly sensitive to the influence of a substituent (to substituent change). 

**Scheme 12:**
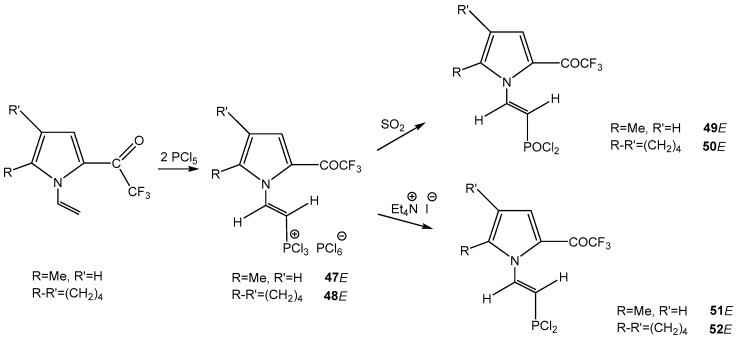
The phosphorylation of 1-vinyl-2-trifluoroacetylpyrroles.

1-vinyl-2-trifluoroacetylpyrroles react with phosphorus pentachloride selectively at the vinyl group to provide *E-*2-(2-trifluoroacetylpyrrol-1-yl)vinylphosphonium hexachlorophosphates **47**, **48,** which further turn into *E*-2-(2-trifluoroacetylpyrrol-1-yl)vinylphosphonyl dichlorides **49**, **50** in almost quantitative yields (Scheme 12, Table 16) [60]. Particularly, in this case, the carbonyl group in the pyrrole ring remains unaffected. 

Similarly, the phosphorus signals in the NMR spectra of salts **47**, **48**, resonating at –297 ppm and 95.7–97.1 ppm, related to PCl_6_^−^ and PCl_3_^+^, correspondently, where the cation signals are observed as doublets ^2^*J*_PH_ = 33 Hz and ^3^*J*_PH_ = 35 Hz, which indicate the *E*-configuration. The proton constant ^3^*J*_HH_ =16 Hz (vinyl group) in the ^1^H NMR spectra also points to the *E*-isomer. 

The NMR data confirm that *E*-isomerism is retained upon passing from complex salts **47**, **48** to the corresponding phosphonic acid dichlorides **49**, **50** and dichlorophosphines **51**, **52**.

**Scheme 13:**
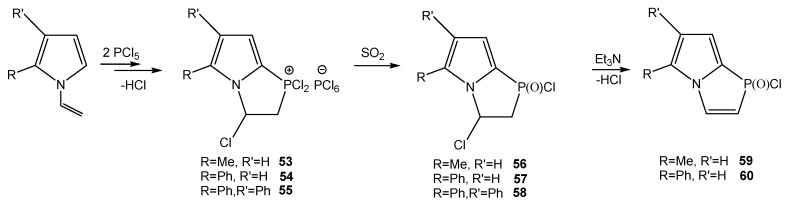
The phosphorylation of 1-vinylpyrroles.

The phosphorylation of *N*-vinylpyrroles which do not contain strong electron-withdrawing groups in the ring, as well as their annulated analogues, *N*-vinylindole and *N*-vinylcarbazole, has been studied [61]. The interaction of these pyrroles with phosphorus pentachloride yields various products of phosphorylation both at the pyrrole ring and at the vinyl group (Scheme 13, Table 17). 

Hexachlorophosphates **53**–**55** after the action of SO_2,_ are converted into the corresponding pyrrolo [1,2-a]-1oxo-1,3-dichloro-4,1-azaphospholanes **56**–**58** (Table 17). The reaction of the salts **53**–**55** and triethylamine proceeds via the elimination of hydrogen chloride to give rise the corresponding phosphol-2-enes **59**, **60**, the structure of which was confirmed by ^1^H, ^13^C and ^31^P NMR spectroscopy data [61].

**Scheme 14:**
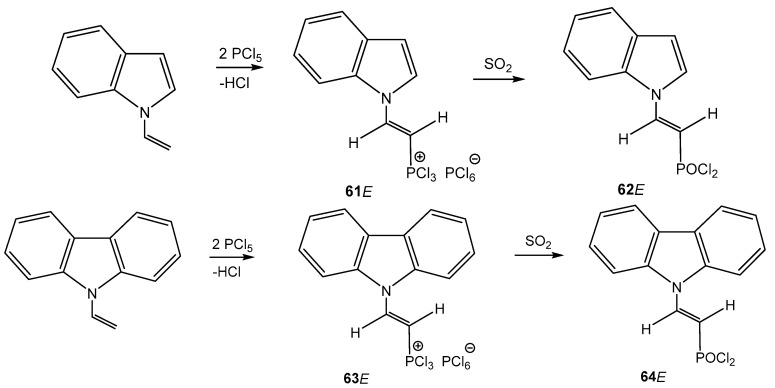
The phosphorylation of 1-vinylindole and 1-vinylcarbazole.

The phosphorylation of indoles and carbazoles occurs exclusively at the vinyl group, as evidenced by the NMR data [61]. The indole and carbazole fragments of the latter are not attacked by phosphorus pentachloride (Scheme 14).

^31^P NMR signals in the spectra of salts **61** and **63** are observed in the region of about –298 ppm and 92–95 ppm, and are assigned to PCl_6_^−^ and PCl_3_^+^, correspondently, thus indicating the *E*-configuration. A proton constant of about ^3^*J*_HH_ =16 Hz in the ethenyl group in the ^1^H NMR spectra also indicates the *E*-isomer. The *E*-isomers are preserved upon passing from salts **61** and **63** to the corresponding phosphonic acid dichlorides **62** and **64**.

## 8. Conclusions

Azoles are the structural core of the most popular drugs used for the treatment of various diseases. Interest in organophosphorus compounds, including those containing azolyl moieties, increases every year due to their high and unusual (specific) biological activity [69,70,71,72,73,74,75,76,77,78,79,80]. In recent years, in connection with the rapid development of experimental technology, previously very problematic studies that are of key importance for the stereochemistry of heterocycles have become rather more possible and routine. This has greatly expanded the possibilities of and increased the information potential of NMR spectroscopy. The use (involvement) of multinuclear NMR spectroscopy, in particular ^31^P, and quantum chemistry (high-level quantum chemical calculations) simplifies the problems associated with stereochemical behavior, for example, Z/E*-*isomerization of chlorophosphorylated *N*-vinylazoles. Increasing the coordination number of phosphorus from 3 to 6 leads to a dramatic shielding of the ^31^P nucleus from about +200 to −300 ppm. Theoretical study of this extraordinary effect in combination (supplement) with experimental data allows a deeper understanding of the nature of the relationship between ^31^P NMR chemical shifts and the structure of organophosphorus compounds with different coordination numbers. ^31^P NMR spectroscopy is the most convenient, powerful and straightforward express method for the recognition of tetra-, penta-, and hexacoordinated phosphorus atoms in phosphorylated *N*-vinylazoles, and for the identification of the stereochemical structure of isomeric forms of organophosphorus products.

## Figures and Tables

**Table 1:** The basicity of substituted azoles.

Compound	pk (BH^+^)	Compound	pk (BH^+^)
1-methylpyrazole	2.06	1-methylimidazole	7.12
1-ethylpyrazole	1.94	1-ethylimidazole	7.19
1-*t*-buthylpyrazole	1.92	1-*n*-propylimidazole	7.16
1,3-dimethylpyrazole	2.77	1-*n*-butylimidazole	7.16
1,4-dimethylpyrazole	2.44	1,2-dimethylimidazole	8.00
1,3,5-trimethylpyrazole	3.74	1-methylbenzimidazole	5.55
1-methylindazole	0.30	1-ethylbenzimidazole	5.62
2-methylindazole	2.01	1-*n*-propylbenzimidazole	5.46
1-methyl-1,2,4-triazole	3.20	1-*n*-butylbenzimidazole	5.31
1-methyl-3-nitro-1,2,4-triazole	−3.51	1-methyl-5-nitrobenzimidazole	3.66
1,3-dimethyl-1,2,4-triazole	3.64	1-methyl-6-nitrobenzimidazole	4.2
1-methyl-1,2,3-triazole	1.23	1-methyl-7-nitrobenzimidazole	3.25
1-methyl-4-bromo-1,2,3-triazole	−1.65	1-methyl-5-chlorobenzimidazole	4.66
1-methyl-5-bromo-1,2,3-triazole	−0.47	benzotriazole	8.38
1-methyl-4-formyl-1,2,3-triazole	−0.58	5(6)-chlorobenzotriazole	7.7
2-methyl-1,2,3-triazole	<1	4,5,6,7-tetrachlorobenzotriazole	5.48

**Table 2:** The ^1^H and ^31^P NMR chemical shifts (δ, ppm) and coupling constants (*J*, Hz) of hexachlorophosphorates **1**–**4**, **9**, **11**, **13**, **18**.

No	Structure	δ^1^H	δ^31^P, *J*_PH_	
			=CH-PCl_3_ 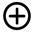	PCl_6_ 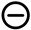
**1** *E*	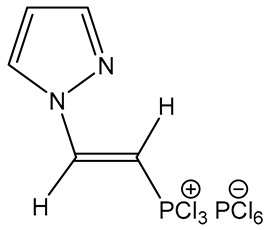	7.96 dd, 1H, =CH-N ^3^*J*_PH_ = 24.8, ^3^*J*_HH_ =14.1 7.64 dd, 1H, =CH-P ^2^*J*_PH_ = 38.5, ^3^*J*_HH_ = 14.1 7.21 d, H-5, ^3^*J*_HH_ = 6.5 7.21 d, H-3, ^3^*J*_HH_ = 5.5 6.1 dd, H-4 ^3^*J*_HH_ = 6.5, ^3^*J*_HH_ = 5.5	94.4 ^2^*J*_PH_ = 38.5 ^3^*J*_PH_ = 24.8	−296.0
**1** *Z*	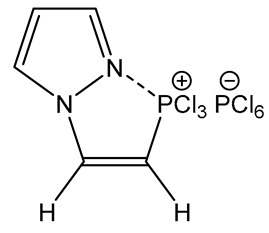	8.26 dd, 1H, =CH-N ^3^*J*_PH_ = 67.7, ^3^*J*_HH_ = 6.1 7.84 dd, =CH-P ^2^*J*_PH_ = 61.4 1H, ^3^*J*_HH_ = 6.1 7.36 d, H-5, ^3^*J*_HH_ = 6.7 7.18 d, H-3, ^3^*J*_HH_ = 5.9 6.1 dd, H-4 ^3^*J*_HH_ = 6.7, ^3^*J*_HH_ = 5.9	−55.2 ^2^*J*_PH_ = 61.4 ^3^*J*_PH_ = 67.7	−295.2
**2** *E*	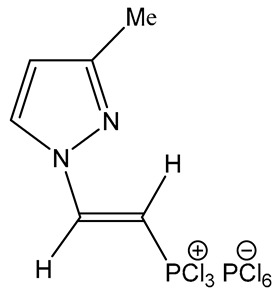	8.02 dd, 1H, =CH-N ^3^*J*_PH_ = 27.5, ^3^*J*_HH_ = 13.3 7.43 dd, 1H, =CH-P ^3^*J*_PH_ = 39.7, ^3^*J*_HH_ = 13.3 7.21 d, H-5, ^3^*J*_HH_ = 5.7 6.48 d, H-4, ^3^*J*_HH_ = 5.7 2.67 s, CH_3_-3	92.9 ^2^*J*_PH_ = 39.7 ^3^*J*_PH_ = 27.5	−296.6
**2** *Z*	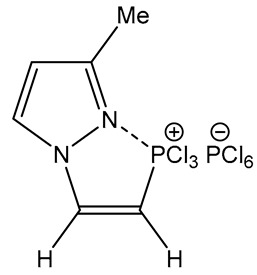	8.34 dd 1H, =CH-N ^3^*J*_PH_ = 62.3, ^3^*J*_HH_ = 6.3 7.42 dd, 1H, =CH-P ^2^*J*_PH_ = 62.3, ^3^*J*_HH_ = 6.3 7.23 s, H-5 6.40 s, H-4 2.68 s, CH_3_-3	−71.4 ^2^*J*_PH_ = 62.3 ^3^*J*_PH_ = 65.7	−295.0
**3** *E*	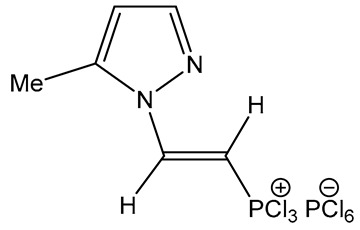	8.40 dd 1H, =CH-N ^3^*J*_PH_ = 27.5, ^3^*J*_HH_ = 15.2 7.66 dd 1H, =CH-P ^2^*J*_PH_ = 39.7, ^3^*J*_HH_ = 15.2 7.11 s, H-3 6.35 s, H-4 2.80 s, CH_3_-5	94.6 ^2^*J*_PH_ = 39.7 ^3^*J*_PH_ = 27.5	−296.0
**3** *Z*	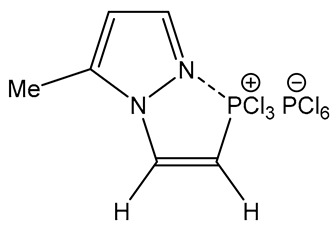	8.44 dd 1H, =CH-N ^3^*J*_PH_ = 67.2, ^3^*J*_HH_ = 6.8 7.73 dd 1H, =CH-P ^2^*J*_PH_ = 61.0, ^3^*J*_HH_ = 6.8 7.32 s, H-3 6.45 s, H-4 2.82 s, CH_3_-5	−39.4 ^2^*J*_PH_ = 61.0 ^3^*J*_PH_ = 67.2	−296.1
**4** *Z*	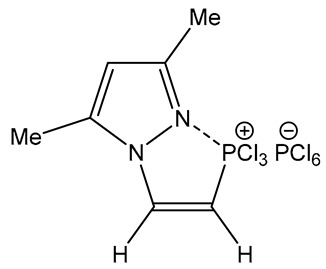	8.54 dd, =CH-N ^3^*J*_PH_ = 64.5, ^3^*J*_HH_ = 6.7 7.63 dd, =CH-P ^2^*J*_PH_ = 60.3, ^3^*J*_HH_ = 6.7 6.41 s, H-4 2.89 s, CH_3_-5 2.76 s, CH_3_-3	−52.9 ^2^*J*_PH_ = 60.3 ^3^*J*_PH_ = 64.5	−296.4
**9** *Z*	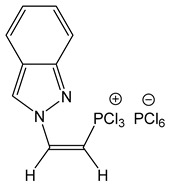	8.27 dd, 1H, =CH-N ^3^*J*_PH_ = 23.8, ^3^*J*_HH_ = 6.8 7.90 s, H-5 7.68 dd, 1H, =CH-P ^2^*J*_PH_ = 35.1, ^3^*J*_HH_ = 6.8 7.39–7.50 4H, Ph	−78.7 ^2^*J*_PH_ = 35.1 ^3^*J*_PH_ = 23.8	−285.0
**11** *E*	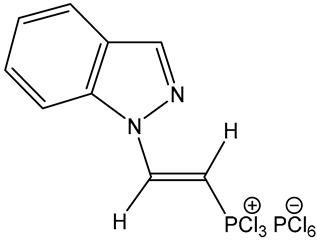	8.16 dd, 1H, =CH-N ^3^*J*_PH_ = 22.8, ^3^*J*_HH_ = 14.1 7.81 s, H-3 7.60 dd, 1H, =CH-P ^3^*J*_PH_ = 34.1, ^3^*J*_HH_ = 14.1 7.24–7.43 4H Ph	73.2 ^2^*J*_PH_ = 34.1 ^3^*J*_PH_ = 22.8	−298.5
**13** *E*	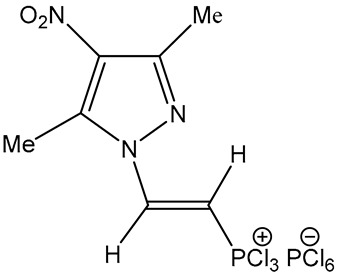	8.47 dd, 1H, =CH-N ^3^*J*_PH_ = 25.8, ^3^*J*_HH_ = 13.4 7.59dd 1H, =CH-P ^2^*J*_PH_ = 38.1, ^3^*J*_HH_ = 13.4 2.78 s, CH_3_-5 2.59 s, CH_3_-3	96.1 ^2^*J*_PH_ = 38.1 ^3^*J*_PH_ = 25.8	−294.9
**18** *E*	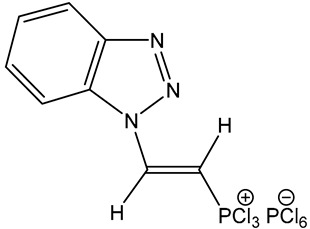	7.84 dd, 1H, =CH-N ^3^*J*_PH_ = 27.6, ^3^*J*_HH_ = 14.4 7.49 dd, 1H, =CH-P ^3^*J*_PH_ = 32.1, ^3^*J*_HH_ = 14.4 7.24–7.43 4H Ph	92.1 ^2^*J*_PH_ = 32.1 ^3^*J*_PH_ = 27.6	−296.2

**Table 3:** The ^31^P NMR chemical shifts (δ, ppm) and coupling constants (*J*, Hz) of hexacoordinated *N*-vinylpyrazoles (**15**–**17**).

Compound	R	R′	δ^31^P	^3^ *J*_PH_	^2^ *J*_PH_
**15**	H	H	−216.2	68.1	55.7
**16**	Me	H	−165.0	68.2	56.1
**17**	Me	Me	−198.8	68.0	58.0

**Table 4:** The ^1^H and ^31^P NMR chemical shifts (δ, ppm) and coupling constants (*J*, Hz) of azolylphosphonates **5**–**8**, **10**, **12**, **14**, **19**.

No	Structure	δ^1^H	δ^31^P
**5** *E*	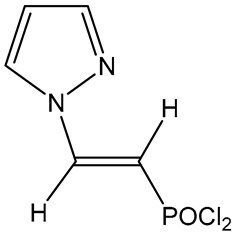	7.71 dd, 1H, N–CH= ^3^*J*_PH_ = 21.8, ^3^*J*_HH_ = 14.2 7.60 d, 1H, H-5 ^3^*J*_HH_ = 5.2 7.23 d, 1H, H-3 ^3^*J*_HH_ = 5.8 6.64 dd, 1H, P–CH= ^2^*J*_PH_ = 28.7, ^3^*J*_HH_ = 14.2 6.15 dd, 1H, H-4 ^3^*J*_HH_ = 5.8, ^3^*J*_HH_ = 5.2	30.7 ^2^*J*_PH_ = 28.7 ^3^*J*_PH_ = 21.8
**5** *Z*	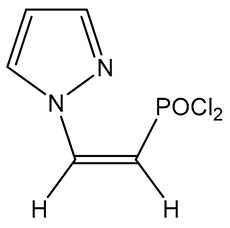	7.57 dd, 1H, N–CH= ^3^*J*_PH_ = 57.0, ^3^*J*_HH_ = 6.7 7.64 d, 1H, H-5, ^3^*J*_HH_ = 5.6 7.21 d, 1H, H-3, ^3^*J*_HH_ = 5.9 6.46 dd, 1H, P–CH= ^2^*J*_PH_ = 28.7, ^3^*J*_HH_ = 6.7 6.12 dd, 1H, H-4 ^3^*J*_HH_ 5.9, ^3^*J*_HH_ 5.6	23.9 ^2^*J*_PH_ = 28.0 ^3^*J*_PH_ = 57.0
**6** *E*	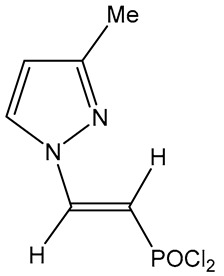	7.75 dd, 1H, N–CH= ^3^*J*_PH_ = 22.0, ^3^*J*_HH_ = 14.5 7.58 d, 1H, H-5, ^3^*J*_HH_ = 5.7 6.63 dd, 1H, P–CH= ^2^*J*_PH_ = 28.5, ^3^*J*_HH_ = 14.5 6.30 d, 1H, H-4, ^3^*J*_HH_ = 5.7 2.35 s, 3H, CH_3_-3	31.3 ^2^*J*_PH_ = 28.5 ^3^*J*_PH_ = 22.0
**6** *Z*	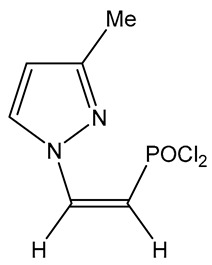	7.81 d, 1H, H-5, ^3^*J*_HH_ = 5.5 7.34 dd, 1H, N–CH= ^3^*J*_PH_ = 57.6, ^3^*J*_HH_ = 11.0 6.60 dd, 1H, P–CH= ^2^*J*_PH_ = 28.5, ^3^*J*_HH_ = 11.0 6.30 d, 1H, H-4, ^3^*J*_HH_ = 5.5 2.33 s, 3H, CH_3_-3	24.4 ^2^*J*_PH_ = 28.5 ^3^*J*_PH_ = 57.6
**7** *E*	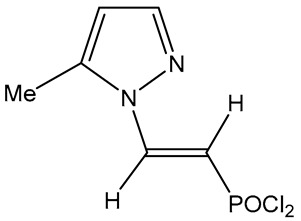	7.64 dd, 1H, N–CH= ^3^*J*_PH_ = 21.4, ^3^*J*_HH_ = 14.4 7.56 d, 1H, H-3, ^3^*J*_HH_ =5.1 6.35 dd, 1H, P–CH= ^2^*J*_PH_ = 28.5, ^3^*J*_HH_ = 14.4 6.12 d, 1H, H-4, ^3^*J*_HH_ = 5.1 2.31 s, 3H, CH_3_-5	32.3 ^2^*J*_PH_ = 28.4 ^3^*J*_PH_ = 21.4
**7** *Z*	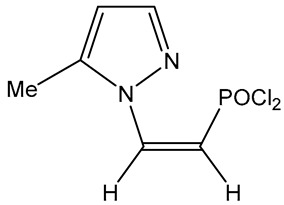	7.53 d, 1H, H-3 ^3^*J*_HH_ = 5.3 7.26 dd, 1H, N–CH= ^3^*J*_PH_ = 54.9, ^3^*J*_HH_ = 10.5 5.73 dd, 1H, P–CH= ^2^*J*_PH_ = 28.8, ^3^*J*_HH_ = 10.5 6.05 d, 1H, H-4, ^3^*J*_HH_ = 5.3 2.34 s, 3H, CH_3_-5	26.8 ^2^*J*_PH_ = 28.8 ^3^*J*_PH_ = 54.9
**8** *E*	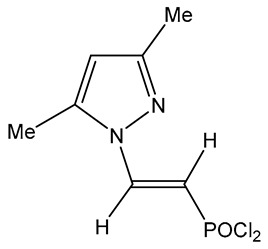	7.59 dd, 1H, N–CH= ^3^*J*_PH_ = 20.8, ^3^*J*_HH_ = 13.7 6.47 dd, 1H, P–CH= ^2^*J*_PH_ = 28.6, ^3^*J*_HH_ = 13.7 5.96 s, 1H, H-4 2.18 s, 3H, CH_3_-5 2.13 s, 3H, CH_3_-3	29.4 ^2^*J*_PH_ = 28.6 ^3^*J*_PH_ = 20.8
**8** *Z*	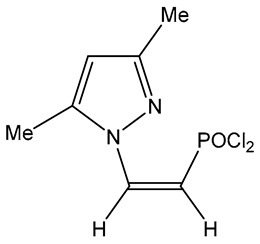	7.24 dd, 1H, N–CH= ^3^*J*_PH_ = 53.7, ^3^*J*_HH_ = 10.3 5.60 dd, 1H, P–CH= ^2^*J*_PH_ = 28.8, ^3^*J*_HH_ = 10.3 5.92 s, 1H, H-4 2.25 s, 3H, CH_3_-5 2.18 s, 3H, CH_3_-3	24.5 ^2^*J*_PH_ = 28.8 ^3^*J*_PH_ = 53.7
**14** *E*	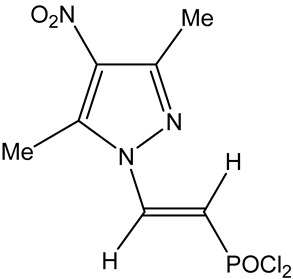	7.74 dd, 1H, N–CH= ^3^*J*_PH_ = 21.5, ^3^*J*_HH_ = 14.0 6.84 dd, 1H, P–CH= ^2^*J*_PH_ = 29.0, ^3^*J*_HH_ = 14.0 2.32 s, 3H, CH_3_-5 2.27 s, 3H, CH_3_-3	26.9 ^2^*J*_PH_ = 29.0 ^3^*J*_PH_ = 21.5
**14** *Z*	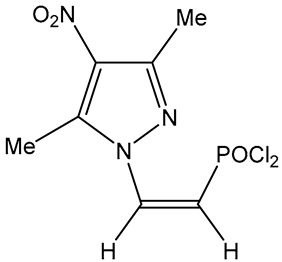	7.59 dd, 1H, N–CH= ^3^*J*_PH_ = 52.9, ^3^*J*_HH_ = 9.0 6.73 dd, 1H, P–CH= ^2^*J*_PH_ = 26.8, ^3^*J*_HH_ = 9.0 2.37 s, 3H, CH_3_-5 2.23 s, 3H, CH_3_-3	26.9 ^2^*J*_PH_ = 26.8 ^3^*J*_PH_ = 52.9
**10** *Z*	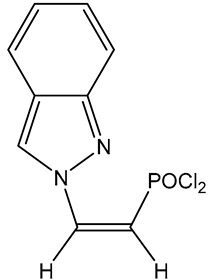	8.44 s, 1H, H-3 7.70 dd, 1H, N–CH= ^3^*J*_PH_ = 56.0, ^3^*J*_HH_ = 10.7 7.20–7.70 m, 4H, Ph 5.98 dd, 1H, P–CH= ^2^*J*_PH_ = 24.9, ^3^*J*_HH_ = 10.7	22.4 ^2^*J*_PH_ = 24.9 ^3^*J*_PH_ = 56.0
**12** *E*	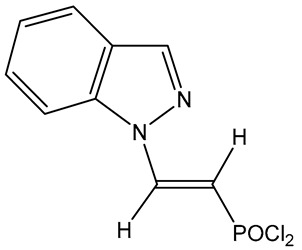	8.24 s, 1H, H-3 8.14 dd, 1H, N–CH= ^3^*J*_PH_ = 27.8, ^3^*J*_HH_ = 14.0 7.30–7.76 m, 4H, Ph 6.63 dd, 1H, P–CH= ^2^*J*_PH_ = 20.5, ^3^*J*_HH_ = 14.0	30.8 ^2^*J*_PH_ = 27.8 ^3^*J*_PH_ = 20.5
**19** *E*	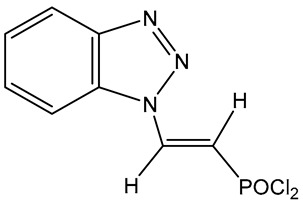	8.26 dd, 1H, N–CH= ^3^*J*_PH_ = 21.8, ^3^*J*_HH_ = 14.9 7.32, 7.51, 7.69, 7.90 4H, Ph 6.91 dd, 1H, P–CH= ^2^*J*_PH_ = 25.2, ^3^*J*_HH_ = 14.9	28.5 ^2^*J*_PH_ = 25.2 ^3^*J*_PH_ = 21.8

**Table 5:** ^31^P NMR chemical shifts (δ, ppm) and coupling constants (*J*, Hz) of hydrochlorated products of azolylphosphonic dichloroanhydrides (**21**–**25**).

Compound	21	22	23	24	25
Structure	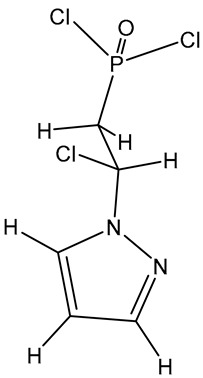	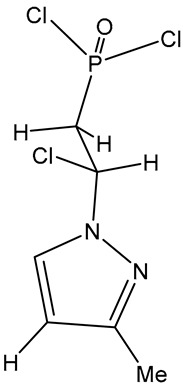	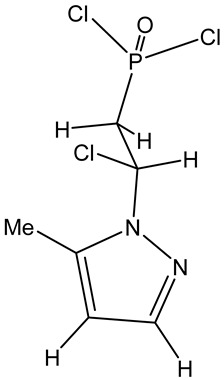	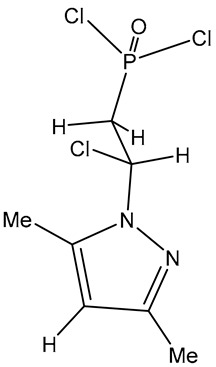	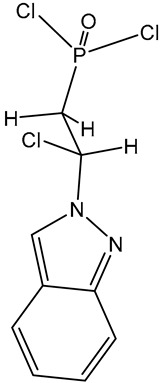
δ, ppm	35.0 ddd	33.5 ddd	34.7 ddd	33.9 ddd	34.2 m
*J*_PH_, Hz	^2^*J* = 17.7 ^2^*J* = 13.5 ^3^*J* = 8.3	^2^*J* = 17.3 ^2^*J* = 13.0 ^3^*J* = 7.9	^2^*J* = 17.8 ^2^*J* = 13.9 ^3^*J* = 8.0	^2^*J* = 17.0 ^2^*J* = 13.2 ^3^*J* = 7.5	-

**Table 6:** Coordination number of phosphorous chlorides and their ^31^P NMR chemical shifts.

Phosphorous Chloride	${\mathrm{PCl}}_{3}$	${\mathrm{PCl}}_{4}^{+}$	${\mathrm{PCl}}_{5}$	${\mathrm{PCl}}_{6}^{-}$
Coordination number	3	4	5	6
^31^P NMR chemical shift, ppm	+217	+80	−80	−298

**Table 7:** The ^31^P NMR chemical shifts of compounds **1** and **15** (ppm) calculated with both nonrelativistic GIAO-B3LYP/IGLO-III and quasi-relativistic ZORA-GIAO-B3LYP/DZP levels.

Medium	Compound	Conformation	B3LYP/ IGLO-III	B3LYP/DZP			Experiment CH_3_NO_2_, 25 °C
				*δ*_NR_	*δ*_SO_	*δ*_R_	
Gas phase	**1** *E*	*s-cis*	145.5	146.5	−34.4	112.1	-
		*s-trans*	147.6	148.5	−34.5	114.0	
	**1** *Z*	*s-cis*	140.2	143.6	−35.2	108.4	-
		*s-trans*	134.2	137.2	−34.7	102.5	
	**1***Z*⋯CH_3_NO_2_	*s-cis*	131.6	136.2	−35.9	100.3	
	**2** *Z*	*s-cis*	−97.2	−91.8	−99.2	−191.0	-
		*s-trans*	33.1	37.7	−73.2	−35.5	
Nitro methane	**1** *E*	*s-cis*	150.7	156.7	−33.7	123.0	94.4
		*s-trans*	151.6	157.0	−34.0	123.0	
	**1** *Z*	*s-cis*	139.8	145.4	−34.6	110.8	−55.2
		*s-trans*	138.3	145.4	−34.4	111.0	
	**1***Z*⋯CH_3_NO_2_	*s-cis*	27.6	34.0	−44.1	−10.1	
	**15** *Z*	*s-cis*	−111.8	−108.7	−89.4	−198.1	−216.2
		*s-trans*	33.0	35.8	−63.8	−28.0	

**Table 8:** ^1^H, ^13^C and ^31^P NMR chemical shifts (δ, ppm) and coupling constants (*J*, Hz) of phosphorylated azoles **28**–**36** (CDCl_3_).

No	Compound	^1^H	^13^C	^31^P
**28**	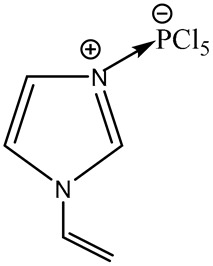	9.27 s, 1H, H-2 8.17 d, 1H, H-5, ^3^*J*_HH_ = 2.0 7.37 d, 1H, H-4, ^3^*J*_HH_ = 2.0 7.06 dd, 1H, =CH, ^3^*J*_HH_ = 15.5 *trans,* ^3^*J*_HH_ =8.7 *cis* 5.70 d 1H =CH_2_, ^3^*J*_HH_ = 15.5 *trans* 5.32 d 1H =CH_2_, ^3^*J*_HH_ = 8.6 *cis*	137.56 C-2 128.81 =CH 126.33 C-4 114.60 C-5 109.77 =CH_2_	−258.0
**29**	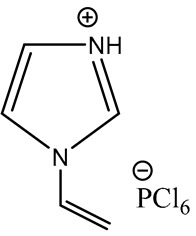	13.3 br s, NH 8.76 s, 1H, H-2 7.65 d, 1H, H-5, ^3^*J*_HH_ = 2.0 7.47 d, 1H, H-4, ^3^*J*_HH_ 2.0 7.13 dd, 1H, =CH, ^3^*J*_HH_ 15.6 *trans,* ^3^*J*_HH_ = 8.8 *cis* 5.76 d, 1H, =CH_2_, ^3^*J*_HH_ = 15.6 *trans* 5.35 d, 1H, =CH_2_, ^3^*J*_HH_ = 8.8 *cis*	134.28 C-2 129.19 =CH 121.33 C-4 119.82 C-5 110.66 =CH_2_	−291.7
**30**	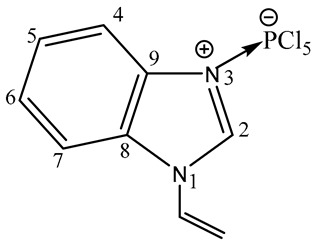	9.18 s, 1H, H-2 7.88 d, 1H, H-7, ^3^*J*_HH_ = 6.8 7.83 d, 1H, H-4, ^3^*J*_HH_ = 7.1 7.62 m, 2H, H-5,6 7.33 dd, 1H, =CH, ^3^*J*_HH_ = 15.8 *trans,* ^3^*J*_HH_ = 8.8 *cis* 5.97 d 1H =CH_2_, ^3^*J*_HH_ = 15.8 *trans* 5.65 d 1H =CH_2_, ^3^*J*_HH_ = 8.8 *cis*	144.28 C-2 139.08 C-9 132.58 C-8 129.08 C-5 123.17 =CH 122.78 C-6 120.31 C-4 119.82 C-7 113.68 =CH_2_	−259.1
**31**	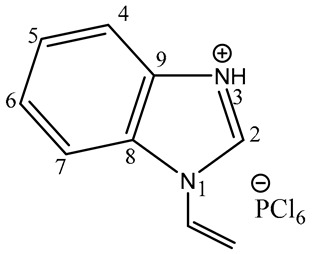	13.6 br s NH 9.16 s, 1H, H-2 7.88 d, 1H, H-7, ^3^*J*_HH_ = 7.2 7.82 d, 1H, H-4, ^3^*J*_HH_ = 7.4 7.66 m, 2H, H-5,6 7.37 dd, 1H, =CH, ^3^*J*_HH_ = 15.8 *trans,* ^3^*J*_HH_ = 8.8 *cis* 5.92 d, 1H, =CH_2_, ^3^*J*_HH_ = 15.8 *trans* 5.68 d, 1H, =CH_2_, ^3^*J*_HH_ = 8.8 *cis*	147.56 C-2 142.38 C-9 134.85 C-8 129.98 C-5 124.27 =CH 122.89 C-6 121.93 C-4 118.18 C-7 114.82 =CH_2_	−293.2
**32**	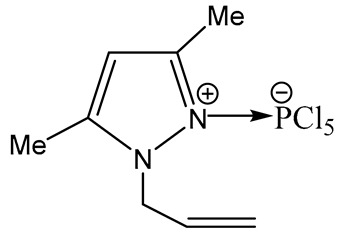	6.70 s, 1H, H-4 6.06 m, 1H, =CH 5.67 d, 1H, =CH_2_, ^3^*J*_HH_ = 15.1 *trans* 5.49 d, 1H, =CH_2_, ^3^*J*_HH_ = 8.2 *cis* 5.24 m, 2H, CH_2_ 2.91 s, 3H, CH_3_-5 2.80 s, 3H, CH_3_-3	145.42 C-3 144.32 C-5 120.83 C-4 119.17 =CH_2_ 107.2 –CH= 49.61 NCH_2_ 10.43 CH_3_ 10.28 CH_3_	−264.3
**32-1**	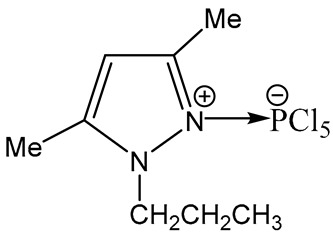	6.33 s, 1H, H-4 4.55 t, 2H, NCH_2_, ^3^*J*_HH_ = 6.8 2.92 s, 3H, CH_3_-5 2.85 s, 3H, CH_3_-3 2.54 m, 2H, CH_2_ 1.63 t, 3H, CH_3_, ^3^*J*_HH_ = 6.6	144.11 C-3 143.40 C-5 109.60 C-4 48.79 NCH_2_ 23.61 CH_2_ 9.83 2CH_3_ 9.54 CH_3_	−261.5
**32–2**	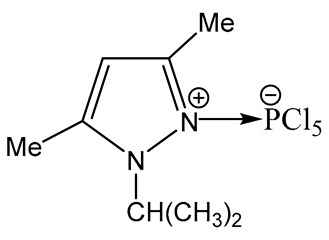	6.11 s, 1H, H-4 4.70 sept, CH, ^3^*J*_HH_ = 6.7 2.80 s, 3H, CH_3_-5 2.76 s, 3H, CH_3_-3 1.59 d, 6H, CH_3_, ^3^*J*_HH_ = 6.6	146.51 C-3 145.79 C-5 107.93 C-4 52.98 CH 21.19 (CH_3_)_2_ 9.65 CH_3_ 9.44 CH_3_	−260.2
**33**	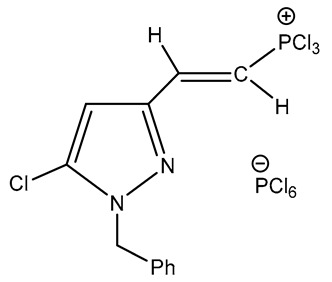	7.50–7.58 Ph 6.77 s, 1H, H-4 6.34 dd, 1H, P–CH= ^2^*J*_PH_ = 58.9, ^3^*J*_HH_ = 15.4 6.23 d, 1H, =CH, ^3^*J*_HH_ = 15.4 5.31 s, 2H, CH_2_	148.09 br s C-5 141.18 C-3 129.20 –CH= 128.8–127.0 Ph 126.9 P–CH=, ^1^*J*_PC_ = 155.1 106.82 C-4 52.60 CH_2_	71.9 d PCl^+^_3_ ^2^*J*_PH_ = 58.9 −295.3 PCl^−^_6_
**34**	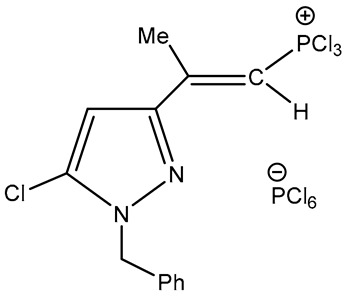	7.50–7.58 Ph 6.77 s, 1H, H-4 6.34 dd, 1H, P–CH=, ^2^*J*_PH_ = 58.9, ^3^*J*_HH_ = 15.4 6.23 s, 1H, =CH, 5.31 s, 2H, CH_2_	150.78 br s C-5 144.49 d, C-CH_3_, ^2^*J*_PC_ = 10.5 133.52 C-3 129.0–127.0 Ph 124.49 =CH, ^1^*J*_PC_ = 153.7 110.78 C-4 57.60 CH_2_ 28.80 d, CH_3_, ^3^*J*_PC_ = 28.9	77.0 d PCl^+^_3_ ^2^*J*_PH_ = 60.3 −294.4 PCl^−^_6_
**35**	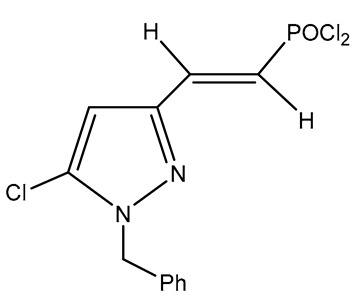	7.30–7.38 Ph 6.70 s, 1H, H-4 6.20 dd, 1H, P–CH= ^2^*J*_PH_ = 29.3, ^3^*J*_HH_ =14.8 6.14 dd, 1H, =CH, ^3^*J*_PH_ = 23.2 ^3^*J*_HH_ = 14.8 5.31 s, 2H, CH_2_	150.91 br s, C-5 135.12 C-3 131.2 –CH= 128.86, 128.20 127.80,127.42 Ph 126.0 d, P–CH=, ^1^*J*_PC_ = 153.1 103.61 C-4 50.61 CH_2_	24.2 dd POCl_2_ ^2^*J*_PH_ = 29.3 ^3^*J*_PH_ = 23.2
**36**	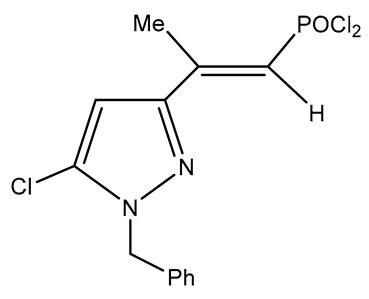	7.28–7.35 Ph 6.79 s, 1H, H-4 6.18 d, 1H, =CH, ^2^*J*_PH_ = 34.3 5.36 s, 2H, CH_2_ 2.34 d, 3H, CH_3,_^4^*J*_PH_ = 1.2	152.01br s C-5 146.99 d, C-CH_3_, ^2^*J*_PC_ = 9.9 135.12 C-3 128.82, 128.24 127.88, 127.51 Ph 121.42 =CH, ^1^*J*_PC_ = 150.4 106.68 C-4 53.56 CH_2_ 24.82 d, CH_3_, ^3^*J*_PC_ = 26.8	28.9 d POCl_2_ ^2^*J*_PH_ = 34.3

**Table 9:** The ^31^P NMR chemical shifts of complexes **28** and **32** calculated by the GIAO-B3LYP/DZP method, taking into accountspin–orbital interaction in comparison with the experimental data.

Medium	Compound	Conformer	Chemical Shift, ppm			
			*δ*_NR_	*δ*_SO_	*δ*_R_	*δ*_exp_
Gas phase	**28**	*s*-*cis*	−69.0	−201.4	−270.4	-
		*s*-*trans*	−57.8	−210.8	−268.6	
	**32**	*s*-*cis*	81.4	−144.5	−63.1	-
		*s*-*trans*	82.3	−145.2	−62.9	
Nitromethane	**28**	*s*-*cis*	−79.7	−181.6	−261.3	−258.0
		*s*-*trans*	−80.2	−181.9	−262.1	
	**32**	*s*-*cis*	−71.0	−204.7	−275.7	−264.3
		*s*-*trans*	−74.9	−207.6	−282.5	

**Table 10:** Experimental and theoretical values of ^31^P NMR chemical shifts of phosphines **5** and **37**–**39** calculated by the GIAO-B3LYP-ZORA/DZP method.

No	Compound	Isomer	Chemical Shift, ppm	
			Calculated ^a^	Experimental
**5**	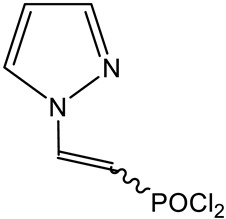	*E*	33.0	31.3
		*Z*	22.0	23.9
**37**	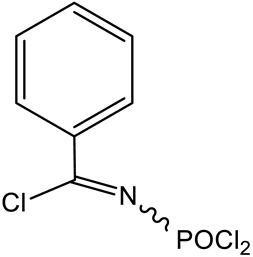	*E*	−3.3	−0.6
		*Z*	−3.1	-
**38**	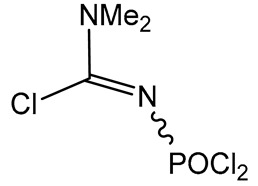	*E*	−14.9	−0.5
		*Z*	−9.8	
**39**	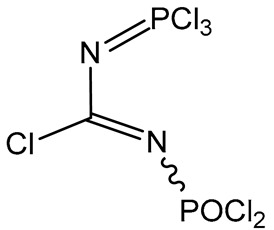	*E*	−2.0 −12.9 = PCl_3_	1.6 22.0 = PCl_3_
		*Z*	−5.7 19.9 = PCl_3_	-

^a^ Averaged by conformational states.

**Table 11:** Theoretical values of ^31^P NMR chemical shifts and relative energies of conformers of the compound **5**.

Structure	Isomer	Conformer	*E*_rel_, kcal/mol	Chemical Shift, ppm
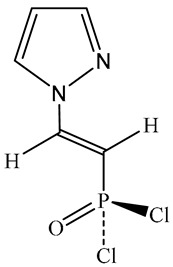	*E*	*s-cis-s-cis*	0.0	33.6
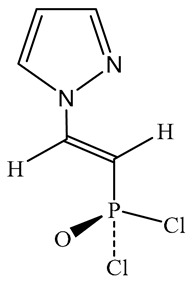	*E*	*s-cis-gauche*	3.1	29.3
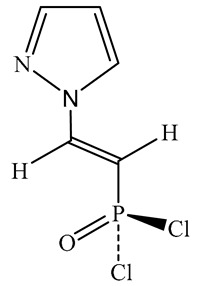	*E*	*s-trans-s-cis*	1.4	30.6
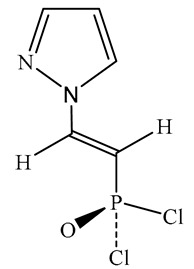	*E*	*s-trans-gauche*	4.0	29.5
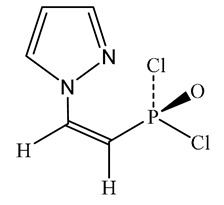	*Z*	*s-cis-gauche*	4.7	21.4
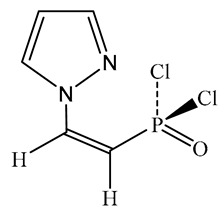	*Z*	*s-cis-s-trans*	6.3	22.9
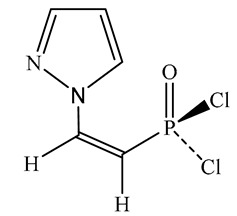	*Z*	*s-trans-s-cis*	6.5	32.8
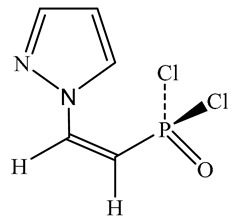	*Z*	*s-trans-s-trans*	6.5	16.6

**Table 12:** Theoretical values of ^31^P NMR chemical shifts and relative energies of conformers **37**.

Structure	Isomer	Conformer	*E*_rel_, kcal/mol	Chemical Shift, ppm
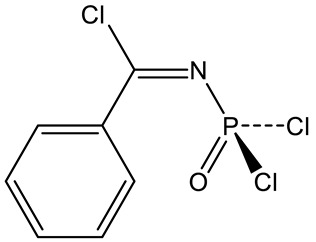	*E*	*s-cis*	5.0	−15.9
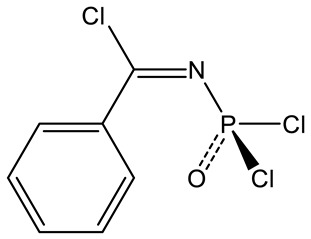	*E*	*gauche*	3.9	−1.3
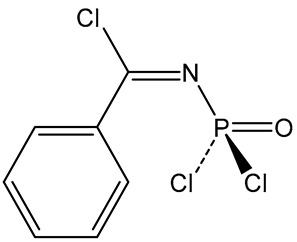	*E*	*s-trans*	8.9	−50.8
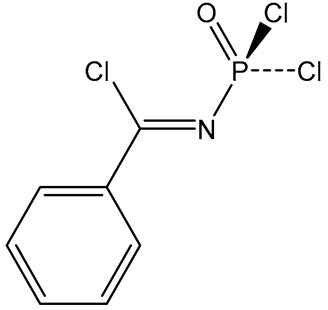	*Z*	*s-cis*	0.5	−1.4
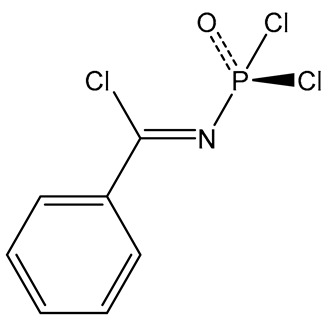	*Z*	*gauche*	0.0	−0.3
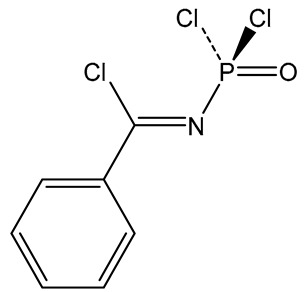	*Z*	*s-trans*	0.7	−14.8

**Table 13:** Theoretical values of ^31^P NMR chemical shifts and relative energies of conformers **38**.

Structure	Isomer	Conformer	*E*_rel_, kcal/mol	Chemical Shift, ppm
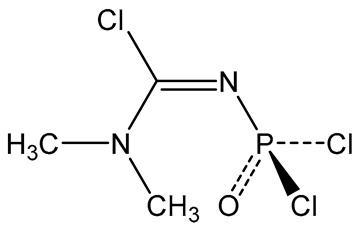	*E*	*gauche*	4.0	−14.9
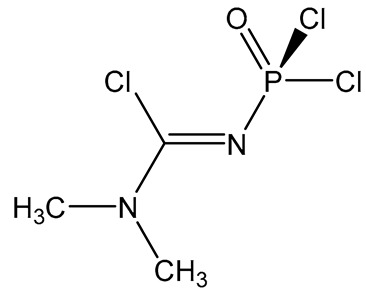	*Z*	*s-cis*	0.5	−5.8
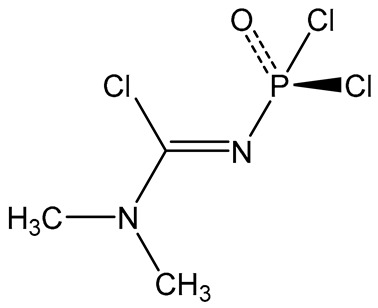	*Z*	*gauche*	0.0	−4.5
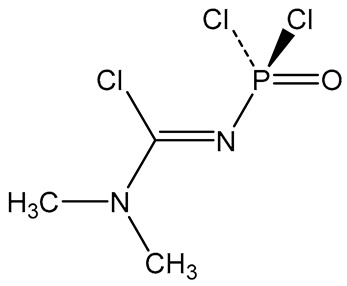	*Z*	*s-trans*	0.3	−21.4

**Table 14:** Theoretical values of ^31^P NMR chemical shifts and relative energies of conformers **39**.

Structure	Isomer	Conformer	*E*_rel_, kcal/mol	Chemical Shift, ppm
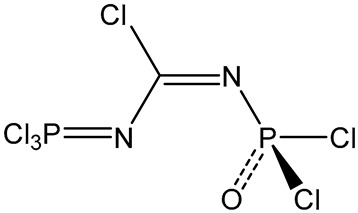	*E*	*s-trans-gauche*	1.8	1.7
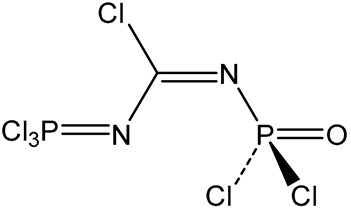	*E*	*s-trans-s-trans*	2.7	−18.7
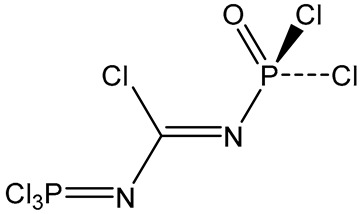	*Z*	*s-trans-s-cis*	3.7	−3.4
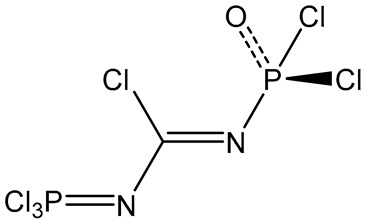	*Z*	*s-trans-gauche*	3.4	−2.4
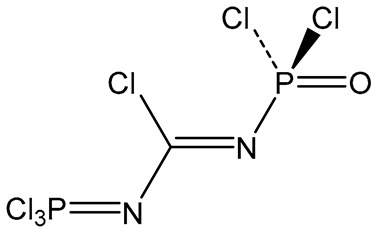	*Z*	*s-trans-s-trans*	4.5	−19.6
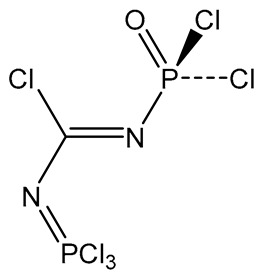	*Z*	*s-cis-s-cis*	0.4	−2.3
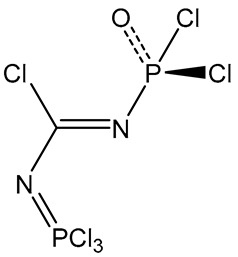	*Z*	*s-cis-gauche*	0.0	−2.1
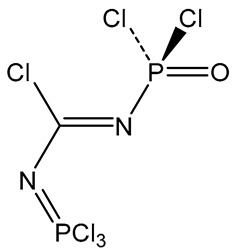	*Z*	*s-cis-s-trans*	0.5	−18.1

**Table 15:** ^1^H, ^13^C and ^31^P NMR chemical shifts (δ, ppm) and coupling constants (*J*, Hz) of phosphorylated pyrroles (DMSO-*d*_6_).

No	Structure	δ ^1^H	δ ^13^C	δ ^31^P
**40**	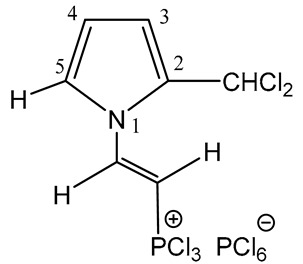	8.31 m, 1H, N–CH= 7.21 m, 1H, H-5 6.82 s, 1H, CHCl_2_ 6.45 m, 1H, H-3 6.23 m, 1H, H-4 6.16 m, 1H, P–CH=	148.7 d, N–CH=, ^2^*J*_PC_ = 37.5 130.9 C-2, 121.4 C-5 114.2 C-3, 112.6 C-4 95.8 d, P–CH=, ^1^*J*_PC_ = 180.0 62.0 CHCl_2_	92.1 PCl_3_^+^ –296.1 PCl_6_^−^
**41**	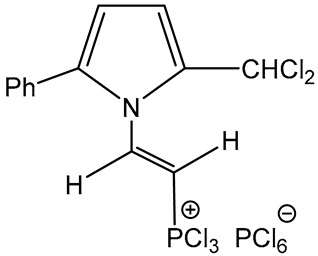	9.11 m, 1H, N–CH= 8.41–8.33 m, 5H, Ph 7.54 m, 1H, H-3 7.22 m, 1H, H-4 7.10 s, 1H, CHCl_2_ 6.59 m, 1H, P–CH=	150.6 d, N–CH=, ^2^*J*_PC_ = 38.0 135.5 C-2, 133.5 C-5 133.0 132.8 131.1 130.9 Ph 119.5 C-3, 118.4 C-4 102.3 d, P–CH=, ^1^*J*_PC_ = 170.0 62.5 CHCl_2_	89.7 PCl_3_^+^ –296.2 PCl_6_^−^
**44**	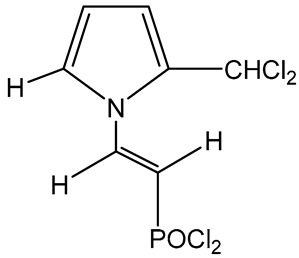	8.22 m, 1H, N–CH= 7.12 m, 1H, H-5 6.80 s, 1H, CHCl_2_ 6.39 m, 1H, H-3 6.20 m, 1H, H-4 6.07 m, 1H, P–CH=	141.3 d, N–CH=, ^2^*J*_PC_ = 21.0 131.5 C-2, 128.7 C-5 114.0 C-4, 112.3 C-3 106.5 d, P–CH=, ^1^*J*_PC_ = 167.0 61.8 CHCl_2_	31.9 POCl_2_
**45**	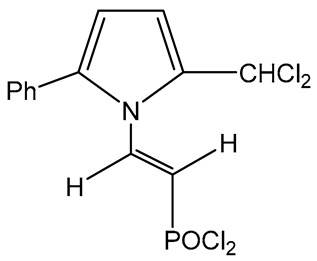	9.87 m, 1H, N–CH= 8.28–7.82 m, 5H, Ph 7.70 m, 1H, H-3 6.97 s, 1H, CHCl_2_ 6.81 m, 1H, H-4 5.73 m, 1H, P–CH=	142.9 d, N–CH=, ^2^*J*_PC_ = 19.9 137.0 C-2, 134.0 C-5 131.5 130.4 129.6 128.0 Ph 124.1 C-3, 117.9 C-4 112.5 d, P–CH=, ^1^*J*_PC_ = 193.8 62.9 CHCl_2_	33.7 POCl_2_
**46**	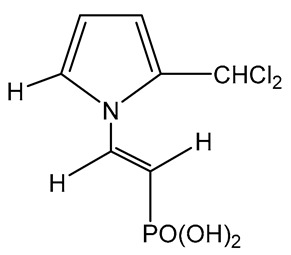	8.26 m, 1H, N–CH= 7.13 m, 1H, H-5 6.77 s, 1H, CHCl_2_ 6.41 m, 1H, H-3 6.27 m, 1H, H-4 6.21 m, 1H, P–CH=	140.9 d, N–CH=, ^2^*J*_PC_ = 25.8 131.7 C-2, 128.8 C-5 114.9 C-4, 111.9 C-3 107.1 d, P–CH=, ^1^*J*_PC_ = 208.0 61.8 CHCl_2_	12.4 PO(OH)_2_

**Table 16:** ^1^H, and ^31^P NMR chemical shifts (δ, ppm) and coupling constants (*J*, Hz) of phosphorylated trifluoroacetylpyrroles (CDCl_3_).

No	Structure	δ ^1^H	δ ^31^P
**49**	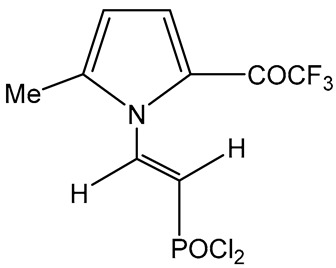	8.59 dd, 1H, N–CH= ^3^*J*_PH_ = 23.3, ^3^*J*_HH_ = 15.6 7.50 s, 1H, H-4 6.59 dd, 1H, P–CH= ^2^*J*_PH_ = 25.7, ^3^*J*_HH_ = 15.6 6.49 s, 1H, H-3 2.61 s, 3H, CH_3_	28.6 ^2^*J*_PH_ = 25.7 ^3^*J*_PH_ = 23.3
**50**	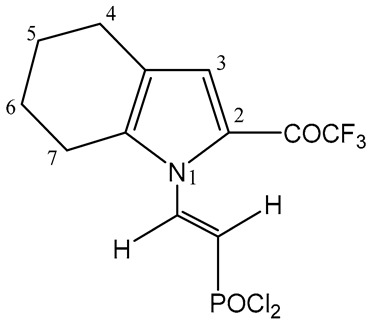	8.72 dd, 1H, N–CH= ^3^*J*_PH_ = 24.2, ^3^*J*_PH_ = 15.7 7.22 s, 1H, H-3 6.59 dd, 1H, P–CH= ^2^*J*_PH_ = 25.9, ^3^*J*_HH_ = 15.7 2.5–2.7 m, 4H, H-4,7 1.6–1.9 m, 4H, H-5,6	29.1 ^2^*J*_PH_ = 25.9 ^3^*J*_PH_ = 24.2
**51**	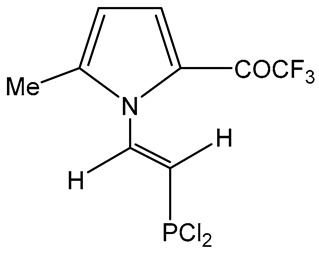	8.50 dd, 1H, N–CH= ^3^*J*_HH_ = 15.3, ^3^*J*_PH_ = 12.5 7.39 s, 1H, H-4 6.42 dd, 1H, P–CH= ^3^*J*_HH_ = 15.3, ^2^*J*_PH_ = 9.4 6.38 s, 1H, H-3 2.59 s, 3H, CH_3_	153.5 ^2^*J*_PH_ = 9.4 ^3^*J*_PH_ = 12.5
**52**	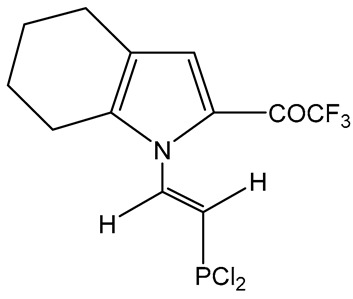	8.28 dd, 1H, N–CH= ^3^*J*_HH_ = 15.5, ^3^*J*_PH_ = 14.5 6.99 s, 1H, H-3 6.46 dd, 1H, P–CH= ^3^*J*_HH_ = 15.5, ^2^*J*_PH_ = 7.3 2.4–2.7 m, 4H, H-4,7 1.7–1.9 m, 4H, H-5,6	160.6 ^2^ *J*_PH_ = 7.3 ^3^*J*_PH_ = 14.5

**Table 17:** ^1^H, and ^31^P NMR chemical shifts (δ, ppm) and coupling constants (*J*, Hz) of phosphorylated pyrroles **56**, **57** (CDCl_3_).

No	Structure	δ ^1^H	δ ^31^P
**56**	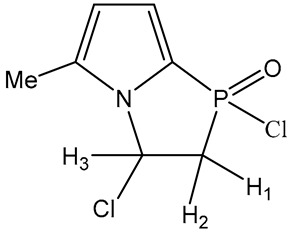	6.68 d, 1H, H-4, ^3^*J*_HH_ = 4.0 6.39 m, 1H, H_3_ 6.19 d, 1H, H-3, ^3^*J*_HH_ = 4.0 3.49 m, 1H, H_1_ 3.17 m, 1H, H_2_ 2.3 s, 3H, CH_3_	35.3 ^2^*J*_PH1_ = 13.5 ^2^*J*_PH2_ = 14.5 ^3^*J*_PH3_ = 27.5
**57**	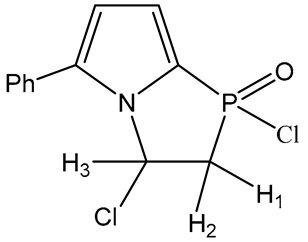	7.4–7.1 m, 5H, Ph 6.75 d, 1H, H-4, ^3^*J*_HH_ = 4.0 6.31 m, 1H, H_3_ 6.15 d, 1H, H-3, ^3^*J*_HH_ = 4.0 3.33 m, 1H, H_1_ 3.05 m, 1H, H_2_	34.6 ^2^*J*_PH1_ = 14.0 ^2^*J*_PH2_ = 14.0 ^3^*J*_PH3_ = 28.0
**59**	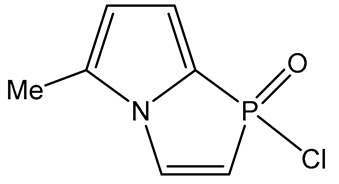	7.38 dd, 1H, N–CH= ^3^*J*_PH_ = 39.2, ^3^*J*_HH_ = 6.1 6.62 dd, 1H, H-3 ^3^*J*_HH_ = 4.2, ^3^*J*_PH-3_ = 2.6 5.84 d, 1H, H-4, ^3^*J*_HH_ = 4.2 5.69 dd, 1H, P–CH= ^2^*J*_PH_ = 17.9, ^3^*J*_HH_ = 6.1 2.07 s, 3H, Me	28.6 ^2^*J*_PH_ = 17.9 ^3^*J*_PH_ = 39.2 ^3^*J*_PH-3_ = 2.6 (H-3)
**60**	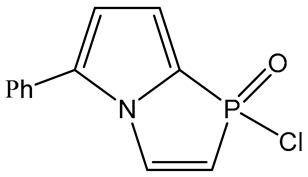	7.4–7.1 m, 5H, Ph 7.38 dd, 1H, N–CH= ^3^*J*_PH_ = 39.5, ^3^*J*_HH_ = 6.5 6.62 dd, 1H, H-3 ^3^*J*_HH_ = 4.2, ^3^*J*_PH-3_ = 2.8 5.84 d, 1H, H-4, ^3^*J*_HH_ = 4.2 5.69 dd, 1H, P–CH= ^2^*J*_PH_ = 18.0, ^3^*J*_HH_ = 6.5	26.4 ^3^*J*_PH_ = 39.5 N–CH= ^2^*J*_PH_ = 18.0 P–CH= ^3^*J*_PH_ = 2.8 (H-3)

## Data Availability

Not applicable.
